# Molecular Diversity and Biochemical Content in Two Invasive Alien Species: Looking for Chemical Similarities and Bioactivities

**DOI:** 10.3390/md21010005

**Published:** 2022-12-22

**Authors:** Julia Vega, Teresa S. Catalá, Jorge García-Márquez, Linn G. Speidel, Salvador Arijo, Niklas Cornelius Kunz, Christoph Geisler, Félix L. Figueroa

**Affiliations:** 1 Andalusian Institute of Blue Biotechnology and Development (IBYDA), Ecology Department, Faculty of Sciences, Malaga University, Campus Universitario de Teatinos s/n, 29071 Malaga, Spain; 2 Research Group for Marine Geochemistry, Institute for Chemistry and Biology of the Marine Environment (ICBM), Carl von Ossietzky University, 26129 Oldenburg, Germany; 3 Organization for Science, Education and Global Society, 70563 Stuttgart, Germany; 4 Andalusian Institute of Blue Biotechnology and Development (IBYDA), Microbiology Department, Faculty of Sciences, Malaga University, Campus Universitario de Teatinos s/n, 29071 Malaga, Spain; 5 Biogeoscience Group, Geological Institute, ETH Zurich, Sonneggstr. 5, 8092 Zurich, Switzerland; 6 Artificial Intelligence in Healthcare and Biotechnology, ValueData GmbH, 51429 Bergisch Gladbach, Germany

**Keywords:** antioxidant capacity, *Asparagopsis armata*, molecular composition, polyphenols, *Rugulopteryx okamurae*, ultrahigh resolution mass spectrometry

## Abstract

The biochemical composition, molecular diversity, and two different bioactivities of *Asparagopsis armata* and *Rugulopteryx okamurae* (two alien species with different invasive patterns in the southern Iberian Peninsula) were analyzed through spectrophotometric methods and Fourier transform ion cyclotron mass spectroscopy (FT-ICR-MS). A total of 3042 molecular formulas were identified from the different extracts. The _d_H_2_O extracts were the most molecularly different. *A. armata* presented the highest content of nitrogenous compounds (proteins, CHON) and sulphur content, whereas *R. okamurae* was rich in carbonated compounds (total carbon, lipids, CHO, and CHOP). Antioxidant capacity and phenolic content were higher in *R. okamurae* than in *A. armata*. Antimicrobial activity was detected from both species. *A. armata* showed capacity to inhibit human and fish pathogens (e.g., *Staphylococcus aureus* or *Vibrio anguillarum*), whereas *R. okamurae* only showed inhibition against human bacteria (*Staphylococcus aureus* and *Cutibacterium acnes*). In *R. okamurae*, molecules with a great number of pharmaceutical activities (e.g., anti-inflammatory or antitumoral), antibacterial, biomaterial, and other utilities were found. The main molecules of *A. armata* had also pharmaceutical applications (e.g., antimalarian, antithrombotic, anti-inflammatory, or antiarthritis). The valorization of these species can help to counteract the environmental effects of the bioinvasions.

## 1. Introduction

Introduced or non-native species are considered one of the major threats to biodiversity, ecosystems, and resource values of the world’s oceans [1,2,3,4]. Invasive species can produce high ecological impacts, modifying natural habitats, trophic relations, and community structures, thereby reducing the growth capacity of native species [5]. Climate change is related to these invasions since the increased temperature is favouring the colonization of exotic species [6]. Bioinvasions also produce socio-economical consequences, which affect the fishing or tourism sectors, among others. The cost of the control, management, and remediation of these bioinvasions is very high [7,8,9]. Seaweeds constitute a dominant group of invasive species, with the Mediterranean Sea being one of the most affected areas [10,11]. Several invasive seaweeds have produced an extreme impact on the marine environment, such as *Undaria pinnatifida* in Europe and Argentina [12,13] or *Ulva prolifera* in the Yellow Sea and East Sea of China [14]. Shipping trade and aquaculture activities have been identified as the main pathways for introducing species in marine environments [15,16,17]. In the Iberian Peninsula, forty-one exotic seaweeds have been recorded [18], although only fifteen species are included in the Spanish catalogue of invasive species [19]. Both species used in this work: *Asparagopsis armata* Harvey (Bonnemaisonilaes, Rhodophyta) and *Rugulopteryx okamurae* ((E.Y.Dawson) I.K. Hwang, W.J. Lee & H.S. Kim) (Dictyotales, Ochrophyta) are included in the catalogue. *R. okamurae*, the most recent, was included in 2020. 

The Rhodophyta *A. armata* is native to southern Australia and New Zealand [20]. Nowadays, this species is distributed in most of the temperate and tropical waters of the world. It is recognized as a globally invasive species and is considered one of the world’s “100 worst invasives” [21]. The introduction of *A. armata* into the Mediterranean Sea is likely to be related to the opening of the Suez Canal and marine transport (fouling or ballast water) [22]. On the Andalusian coast, it can be found from Cadiz to Almeria, forming natural vegetation belts on exposed coasts. The genus *Asparagopsis* presents a triphasic life cycle with a haploid gametophyte and a diploid filamentous tetrasporophyte (named *Falkenbergia*, that in the first instance was considered a separate species) [23]. Tetrasporophytes can be dispersed through marine currents, releasing haploid tetraspores that will form new thallus in different areas. 

In the last years, Ochrophyta *R. okamurae* has been causing a drastic invasion on the Andalusian coast. In 2015, *R. okamurae* was detected on the coast of Ceuta and northern Morocco [24] and in the Strait of Gibraltar [25,26]. One year later, *R. okamurae* covered most of the rocky areas from 0 to 40 m depth in the Strait of Gibraltar [27,28]. This species presents vegetative propagules, tetra, and monospores, which explains the rapid propagation of this species in the area [25]. Northern Africa and the southern Iberian Peninsula constitute the most intensely affected area by the brown alga, which continues its expansion westward and eastward with a trend to monopolize the sea rocky bottom to the detriment of the photophilous resident biota. This species can be floating and transported by the horizontal and vertical currents from the hot point of the Gibraltar Strait until far-extending areas [28], colonizing them or producing high-density beach cast events. *R. okamurae* is drastically reducing the biodiversity [29,30] of an area with high ecological status, such as the Atlantic coast [31,32] and the Mediterranean Sea [33]. The first citation of this species in Europe was in 2002 in the Thau lagoon (France), associated with oyster aquaculture [34]. In the Strait of Gibraltar, it is believed to have been introduced through ballast waters due to high marine trade in the area (Algeciras (Spain) and TangerMed, (Morocco) harbors) [28]. 

Forming algae blooms and invasive species can be used as a biomass source for the extraction of bioactive compounds for different applications [18,35,36]. This can be a strategy to obtain unique bioactive compounds from highly available biomass and, therefore contribute to the mitigation of the negative effects caused by the alien species through the collection. Over the last decades, the red algae, *A. armata* have been studied for their capacity to produce bioactive compounds, with the main results pointing towards their high potential as producers of antioxidant [37,38,39], antifouling [40], antimicrobial [41,42,43], or antiviral [44] compounds. However, in *R. okamurae,* there are only a few publications on biochemical composition or bioactivities. Terpenoids are almost the sole molecules studied in *R. okamurae*, as they are interesting target compounds for being exploited by the industry as feeding deterrents, antifungals, antibiotics, anti-inflammatories, insecticides, or antivirals [45,46,47,48,49,50,51]. Metabolomics studies can help to elucidate the presence of new molecules with promising applications in those species. 

The aim of this study was (1) to analyze the biochemical composition, molecular diversity, and bioactivities of both invasive species; (2) to evaluate the similarities and differences in compounds possibly related to the invasion success; and (3) to determine promising targeted molecules for miscellaneous biotechnological applications. 

## 2. Results

### 2.1. Biochemical Composition

The content of organic matter, ashes, total internal carbon, nitrogen, and sulphur, as well as the carbohydrates, proteins, and lipids content are shown in Table 1. All variables analyzed showed significant differences between both species (Student’s *t*-test, *p* < 0.05).

The organic matter was higher in *Ruguloteryx okamurae* than in *Asparagosis armata* (72.2 and 58.2%, respectively), whereas ashes were higher in *A. armata* than in *R. okamurae* (41.8 and 27.8%, respectively). The different compounds can be divided into carbonated or nitrogenous. *R. okamurae* presented a higher C content, whereas *A. armata* presented a higher N percentage. Therefore, the C:N ratio was much higher in *R. okamurae*. *A. armata* also presented the highest content of total sulphur, total proteins, and carbohydrates, whereas *R. okamurae* presented the highest lipids content. 

**Figure 1:**
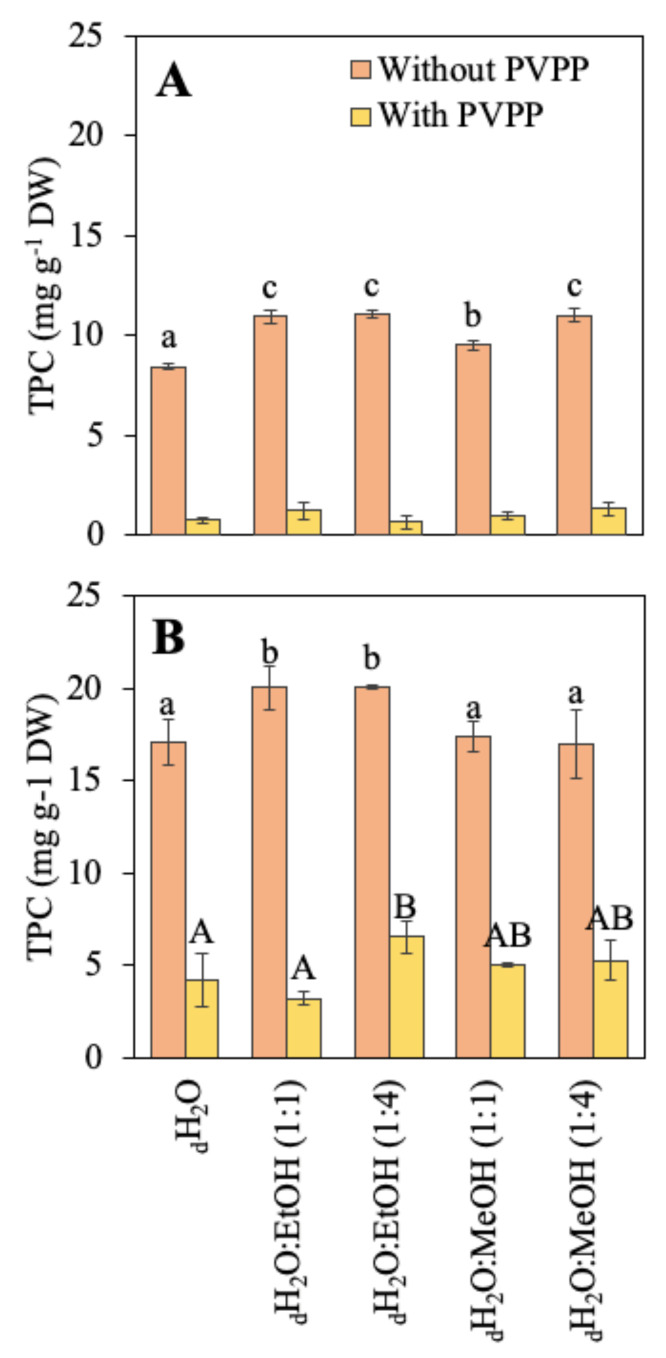
Total phenolic compounds (TPC) content before and after the use of PVPP (mg g^−1^ DW) of the different extractions in different solvents, including water (_d_H_2_O), _d_H_2_O:Ethanol (EtOH) (1:1), _d_H_2_O:EtOH (1:4), _d_H_2_O:Methanol (MeOH) (1:1), and _d_H_2_O:MeOH (1:4), from the biomass of (**A**) *Asparagopsis armata* and (**B**) *Rugulopteryx okamurae*. Values are expressed as average ± standard deviation (SD) (*n* = 3). Different letters indicate significant differences among solvents for each species (ANOVA, *p* < 0.05, SNK test).

The total phenolic compounds (TPC) of both species are shown in Figure 1. Different solvents (aqueous and hydroalcoholic) were compared for the extraction of these molecules and PVPP was used to obtain a more accurate content. *R. okamurae* presented a higher phenolic compound content than *A. armata*. In *R. okamurae,* the highest content was obtained when the extraction was made in the two combinations of _d_H_2_O:EtOH solvents. After the use of PVPP, TPC content drastically decreased (around 68–84%, depending on the solvent used). In *A. armata*, TPC content was highest in the two combinations of _d_H_2_O:EtOH and in _d_H_2_O:MeOH (1:4). In this case, the decrease in the phenol content after the use of PVPP was much higher (around 88–94%, depending on the solvent used).

### 2.2. Molecular Diversity and Published Bioactivities

**Figure 2:**
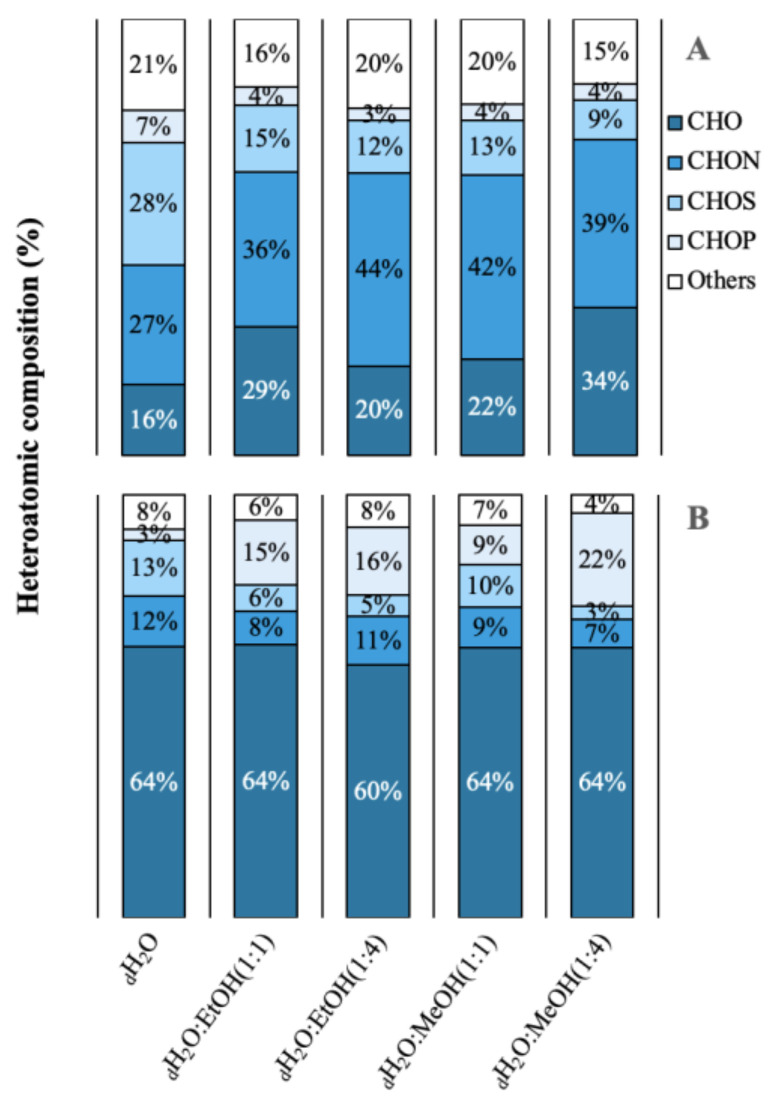
Heteroatomic composition in relative abundance of the different extractions in different solvents of water (_d_H_2_O), _d_H_2_O:Ethanol (EtOH) (1:1), _d_H_2_O:EtOH (1:4), _d_H_2_O:Methanol (MeOH) (1:1), _d_H_2_O:MeOH (1:4), from the biomass of (**A**) *Asparagopsis armata* and (**B**) *Rugulopteryx okamurae*.

**Figure 3:**
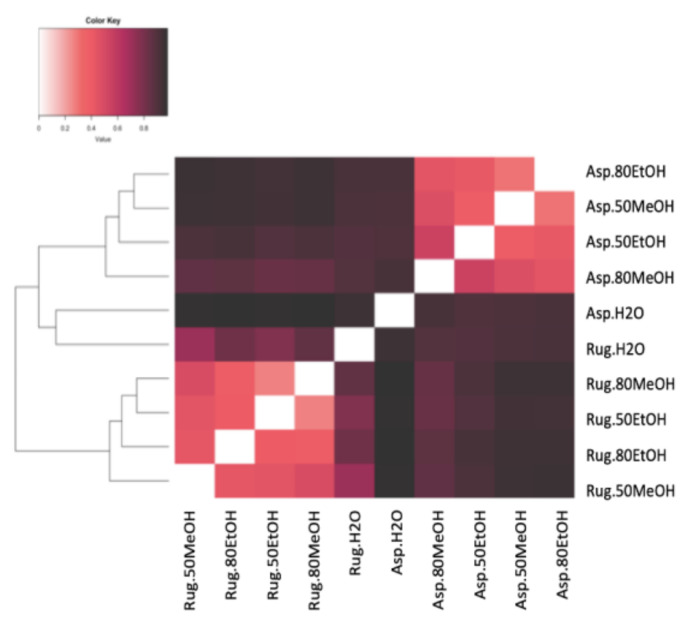
Bray–Curtis dissimilarity analyses of the different extracts in different solvents of water (_d_H_2_O), _d_H_2_O:Ethanol (EtOH) (1:1), _d_H_2_O:EtOH (1:4), _d_H_2_O:Methanol (MeOH) (1:1), and _d_H_2_O:MeOH (1:4), from the biomass of Asparagopsis armata (Asp) and Rugulopteryx okamurae (Rug). All molecular formulas detected in the different samples via FT-ICR-MS signal intensities were included, with black representing 100% similar and white 100% dissimilar.

A total of 3042 molecular formulas were identified from the extracts of both species. In Figure 2, the formulas are grouped according to their heteroatomic composition. In *A. armata*, the CHON content exceeded the CHO content in all extracts, with a maximum of 44% and 42% in _d_H_2_O:EtOH (1:4) and _d_H_2_O:MeOH (1:1), respectively. The percentage of CHO compounds varied from 16 to 34% and of “others” from 15 to 21%. The presence of CHOS was also significant, with a maximum of 28% in the aqueous solution. CHOP content was minimal in this species. *R. okamurae* presented a majority of CHO formulas in all extracts, with more than 50% of the total number of formulas. In this species, CHON formulas are less abundant, ranging from 7 to 12%. Contrary to *A. armata*, CHOP formulas are more abundant in this species, ranging from 9% in _d_H_2_O:MeOH (1:1) to 22% in _d_H_2_O:MeOH (1:4). Table S1 shows the percentages of the different molecular groups. *A. armata* extracts were more enriched in unsaturated formulas (with nitrogen -N- and oxygen -O- poor), especially using _d_H_2_O:EtOH (1:1), _d_H_2_O:EtOH (1:4), and _d_H_2_O:MeOH (1:4). This species also showed a high aromatic content (O poor) in the aqueous extract (26 ± 4%). Saturated formulas were scarce all over the extracts, with a maximum of 4% in *A. armata* _d_H_2_O:EtOH (1:1). *R. okamurae* extracts were more enriched in highly unsaturated formulas (O poor), exceedingly even 50% of the total molecular formulas in three of the extracts. Unsaturated (O poor) and aromatic (O poor) were the next more abundant groups in *R. okamurae* (19–35% and 10–20%, respectively). The _d_H_2_O extracts were the most molecularly different in the two species (Figure 3), showing the highest number of exclusive formulas (Table S1).

The molecular formulas with the highest intensities were further investigated for the two species with the software *ChemCrawler.* Only those formulas with identified bioactivities associated with an isomer of natural origin are shown (Table 2 and Table 3). Those isomers of artificial synthesis were discarded. 

In *A. armata* (Table 2), three molecules (two chromones and one trihydroxyoctadecenoic acid) with anti-inflammatory activity and two with analgesic properties were found in _d_H2O and _d_H_2_O:MeOH(1:4) [52,53,54]. Multiple other health benefits were observed in the formula C_22_H_28_O_11_ in the form of chromones [55,56,57,58]. Metabolites with astringent properties, iron chelators, nephroprotective agents, and members of the human blood serum were also detected [59]. A non-essential amino acid with a nutraceutical, protective, and micronutrient role has also been identified in _d_H_2_O:EtOH(1:1) [60]. The sweetener maltotriose (C_18_H_32_O_16_) has also been found in three extracts [61,62,63]. The second top isomer of the same molecular formula C_18_H_32_O_16_ was associated with dextran, a natural product with an anti-thrombotic effect [64] and bread texture improver [65]. A sesquiterpene lactone (C_20_H_24_O_8_) with anti-malarial activity [66] and a phenolic (C_23_H_30_O_11_) with anti-tobacco mosaic virus (TMV) activity [67] were identified in _d_H_2_O:MeOH(1:4). Lastly, ascorbic acid (Vitamin C) was also detected in _d_H_2_O:MeOH(1:4) [68]. 

In *R. okamurae* (Table 3), the majority of the most abundant molecular formulas were present in all extracts, except the aqueous extract, which was the most molecularly different. Among those, two prostaglandins were identified (C_20_H_34_O_5_ and C_20_H_34_O_6_) with roles as an oxytocic, luteolytic, abortifacient, and vasodilator agents [69,70], together with a hydroxycoumarin (C_10_H_8_O_5_) with multiple medical applications [71,72,73]. Multiple sesquiterpenes were also present with the molecular formulas C_24_H_34_O_10_, C_20_H_24_O_8_, C_19_H_28_O_6_, C_17_H_20_O_7_, C_15_H_18_O_4_, and C_15_H_18_O_3_, with a myriad of benefits, namely mycotoxic, anti-malarial, anti-diabetic, anti-inflammatory, anti-tumoral, anti-allergenic, anti-fungal, and anti-bacterial activity, among others [66,74,75,76,77,78,79]. A coumarin (C_15_H_16_O_3_) with antioxidant, immunomodulatory, and estrogen-like properties was observed in all extracts, other than the aqueous [80,81,82]. 

As both species present a relevant content of polyphenols, a search for common polyphenols was reported. Twenty-eight polyphenols were found in both species, although only those with the highest intensities and clear bioactivities are shown (Table 4). Various molecules with antioxidant, anti-cancer, and anti-inflammatory activities were identified [83,84,85,86,87]. Anti-bacterial activity against *Staphylococcus epidermidis* and *Staphylococcus aureus* [88], anti-leishmanial agent [89], natural UV-A filtering agent [90], among other bio-activities, were also associated to the top isomers of the common polyphenols. 

A search for common terpenoids was also done. A total of six monoterpenes, namely C_10_H_16_O, C_10_H_16_O_2_, C_10_H_16_O_3_, C_10_H_16_O_4_, C_10_H_16_O_5_, and C_10_H_16_N_2_O_2_, and four sesquiterpenes, namely C_15_H_24_O_2_, C_15_H_24_O_3_, C_15_H_24_O_4_, and C_15_H_24_O_6_, were identified in both species. Other sesquiterpenes were species-specific, and some of them were even found in high amounts, mostly in *R. okamurae* (Table 2 and Table 3). Monoterpenes and sesquiterpenes with sulphur content were only present in *A. armata* in the form of C_10_H_16_O_10_S, C_10_H_16_O_11_S, C_10_H_16_O_12_S, and C_14_H_24_O_15_S. Diterpenoids (C_20_H_32_O) and sesterpenes (C_25_H_40_O_9_, C_25_H_40_O_10_) were only present in *R. okamurae*.

**Table 2:** Identification of possible top isomers of natural origin, molecular types, and associated bio-activities from the main formulas found in the different extraction solvents of water (_d_H_2_O), _d_H_2_O:Ethanol (EtOH) (1:1), _d_H_2_O:EtOH (1:4), _d_H_2_O:Methanol (MeOH) (1:1), and _d_H_2_O:MeOH (1:4), from the biomass of *Asparagopsis armata*.

Abbreviation	Formulas	Top Isomer(s)	Molecule Iype	Bio-Activities	Ref.
_d_H_2_O	C_18_H_34_O_5_	Pinellic acid	Trihydroxyoctadecenoic acid	Adjuvant and an anti-inflammatory agent	[53]
		9,10,13-TriHOME	Monounsaturated fatty acid	It has a role as a human blood serum metabolite	[91]
_d_H_2_O:EtOH(1:1) _d_H_2_O:EtOH(1:4) _d_H_2_O:MeOH(1:1)	C_18_H_32_O_16_	Maltotriose	Trisaccharide	Sweetener produced from starch Used in baked goods, and beer and other fermented drinks production Prebiotic effect	[61,62,63]
		Dextran	Glucan	Anti-thrombotic effect, blood viscosity reducer and a volume expander Bread texture improvers	[64,65]
_d_H_2_O:EtOH(1:1) _d_H_2_O:EtOH(1:4) _d_H_2_O:MeOH(1:1)	C_10_H_18_N_2_O_3_	Dethiobiotin	Conjugate acid	Agonists of nuclear receptor subfamily 2, group E, member 3 (NR2E3)	[92]
_d_H_2_O:EtOH(1:1)	C_8_H_12_N_2_O_2_	Pyridoxamine	Monohydroxypyridine	Human, *Saccharomyces cerevisiae*, *Escherichia coli*, plant, and mouse metabolite, an iron chelator, and a nephroprotective agent	[93]
_d_H_2_O:EtOH(1:1)	C_7_H_6_O_5_	Gallic acid	Phenolic	Astringent, a cyclooxygenase 2 inhibitor, a plant metabolite, antioxidant, antineoplastic agent, a human xenobiotic metabolite, an EC 1.13.11.33 (arachidonate 15-lipoxygenase) inhibitor, an apoptosis inducer, and a geroprotector	[59]
_d_H_2_O:MeOH(1:1)	C_6_H_13_N_3_O_3_	Citrulline	Non-essential amino acid	EC 1.14.13.39 (nitric oxide synthase) inhibitor, a protective agent, a nutraceutical, a micronutrient, and a human, an *Escherichia coli*, a *Saccharomyces cerevisiae,* and a mouse metabolite	[60]
_d_H_2_O:MeOH(1:4)	C_22_H_28_O_11_	Prim-O-glucosylcimifugin	Chromone	Anti-pyretic, analgesic and anti-inflammatory activities Used in many Kampo prescriptions Anti-rheumatoid, anti-inflammatory, immunosuppressive, and pain-relieving properties	[52,55,56,58,94,95,96]
		4′-O-Glucosyl-5-O-methylvisamminol	Chromone	Analgesic, anti-inflammatory, anti-psoriasis, and antiplatelet aggregation effects	[54,57]
_d_H_2_O:MeOH(1:4)	C_23_H_30_O_11_	Yadanzioside D		Anti-Tobacco Mosaic Virus (TMV) activity	[67,97]
_d_H_2_O:MeOH(1:4)	C_20_H_24_O_8_	Vernodalol	Sesquiterpene lactone	Angeloylated germacranolides from *Daucus virgatus* and their plasmodium transmission blocking activity	[66]
_d_H_2_O:MeOH(1:4)	C_6_H_8_O_6_	Ascorbic acid (Vitamin C)	Natural water-soluble vitamin	Potent reducing and antioxidant agent found in citrus and other fruits, and in vegetables	[68]

**Table 3:** Identification of possible top isomers of natural origin, molecular types, and associated bio-activities from the main formulas found in the different extraction solvents of water (_d_H_2_O), _d_H_2_O:Ethanol (EtOH) (1:1), _d_H_2_O:EtOH (1:4), _d_H_2_O:Methanol (MeOH) (1:1), and _d_H_2_O:MeOH (1:4), from the biomass of *Rugulopteryx okamurae*.

Abbreviation	Formulas	Top Isomer(s)	Molecule Type	Bio-Activities	Refer.
_d_H_2_O	C_10_H_8_O_5_	Fraxetin	Hydroxycoumarin	Antimicrobial agent, apoptosis inhibitor, apoptosis inducer, antioxidant, anti-inflammatory agent, a hepatoprotective agent, antibacterial agent, and a hypoglycemic agent	[71,72,73]
_d_H_2_O	C_20_H_34_O_5_	Prostaglandin F2alpha	Prostaglandin	Oxytocic, luteolytic, abortifacient, and vasocontractile activities	[69]
		Alprostadil	Prostaglandin	Potent vasodilator agent that increases peripheral blood flow, inhibits platelet aggregation, and induces bronchodilation	[70]
_d_H_2_O	C_18_H_28_O_3_	12-Oxo-phytodienoic acid	Unsaturated fatty acid	Inhibitor of the protein serine/threonine kinase 33 (STK33)	[98]
_d_H_2_O	C_20_H_34_O_6_	Thromboxane B2	Stable metabolite	Human and mouse metabolite	[99]
		6-Keto-prostaglandin F1alpha	Prostaglandin	Human and mouse metabolite	[100]
_d_H_2_O:EtOH(1:1) _d_H_2_O:MeOH(1:1) _d_H_2_O:MeOH(1:4)	C_20_H_24_O_8_	Vernodalol	Sesquiterpene lactone	Angeloylated germacranolides from *Daucus virgatus* and their plasmodium transmission blocking activity	[66]
_d_H_2_O:EtOH(1:1) _d_H_2_O:EtOH(1:4) _d_H_2_O:EtOH(1:4) _d_H_2_O:MeOH(1:4)	C_17_H_22_O_4_	1-Dehydro-[6]-gingerdione	Hydroxycinnamic acid	Antiallergic potential	[101]
_d_H_2_O:EtOH(1:1) _d_H_2_O:MeOH(1:1) _d_H_2_O:MeOH(1:4)	C_14_H_16_O_5_	1′-Acetoxyeugenol acetate	Phenylpropanoid	Anti-breast cancer properties	[102]
_d_H_2_O:EtOH(1:1) _d_H_2_O:MeOH(1:1)	C_12_H_14_O_2_	Precocene I	Chromene, aromatic ether	Member of precocenes and a plant metabolite	[103]
_d_H_2_O:EtOH(1:1) _d_H_2_O:MeOH(1:4)	C_12_H_14_O_3_	Acetyleugenol	Phenol, benzoate ester	Antivirulence potential against pathogenic bacteria of medical importance	[104]
_d_H_2_O:EtOH(1:1) _d_H_2_O:MeOH(1:1) _d_H_2_O:MeOH(1:4)	C_24_H_34_O_10_	3. ′-Hydroxy-T2 Toxin	Sesquiterpene	A trichothecene with mycotoxin effects	[79,105]
_d_H_2_O:EtOH(1:1) _d_H_2_O:MeOH(1:4)	C_19_H_28_O_6_	Tirotundin	Sesquiterpene lactone	Antidiabetic effect through PPARγ pathway	[106]
_d_H_2_O:EtOH(1:1) _d_H_2_O:MeOH(1:1) _d_H_2_O:EtOH(1:4) _d_H_2_O:MeOH(1:4)	C_19_H_24_O_3_	2-Methoxyestrone	Steroid	Indicator of the risk of prostate, colorectal, and breast cancer	[107,108,109]
_d_H_2_O:EtOH(1:1) _d_H_2_O:MeOH(1:1) _d_H_2_O:EtOH(1:4) _d_H_2_O:MeOH(1:4)	C_15_H_18_O_4_	Helenalin	Sesquiterpene lactone	Anti-inflammatory and anti-neoplastic agent Anti-tumoral effects	[75,110,111]
		Parthenin	Sesquiterpene lactone	It is genotoxic, allergenic, and an irritant It is believed to be responsible for the dermatitis caused by *Parthenium hysterophorus*	[77,112]
_d_H_2_O:EtOH(1:1) _d_H_2_O:MeOH(1:1) _d_H_2_O:EtOH(1:4) _d_H_2_O:MeOH(1:4)	C_15_H_16_O_3_	Osthole	Coumarin	Antioxidant and immunomodulatory properties, commonly applied in clinical practice of Traditional Chinese Medicine for cancer, inflammation, etc. It has estrogen-like effects that prevent osteoporosis and reduce bone loss in ovariectomized rats by activation of β-catenin-BMP signaling It is a chromatin regulator implicated in the inhibition of histone deacetylases (HDACs) in order to cure or prevent cancer	[80,81,82,113]
		Batatasin III	Stilbenoid	Anti-inflammatory and anti-cancer activity	[114,115]
_d_H_2_O:EtOH(1:1) _d_H_2_O:EtOH(1:4) _d_H_2_O:MeOH(1:1) _d_H_2_O:MeOH(1:4)	C_15_H_18_O_3_	Santonin	Propionic acid	Effective for treating intestinal roundworms	[116,117]
		Irofulven	Sesquiterpene	A natural toxin with anti-cancer potential isolated from the fungus *Omphalotus illudens*	[59]
		Xanthatin	Sesquiterpene lactone	Tumor suppressor function	[78]
_d_H_2_O:EtOH(1:1) _d_H_2_O:EtOH(1:4) _d_H_2_O:MeOH(1:1) _d_H_2_O:MeOH(1:4)	C_17_H_20_O_7_	Dihydroscandenolide	Sesquiterpene lactone	Anti-fungal and anti-bacterial activity	[74]
		Yomogiartemin	Sesquiterpene lactone	Anti-malarial activity	[76]
_d_H_2_O:EtOH(1:4) _d_H_2_O:MeOH(1:1)	C_23_H_34_O_7_	Nargenicin A1	Saturated alicyclic polyketide	Bacterial macrolide Anti-cancer activity, immunomodulation, and cell protective effect	[118]
_d_H_2_O:EtOH(1:1) _d_H_2_O:EtOH(1:4) _d_H_2_O:MeOH(1:1) _d_H_2_O:MeOH(1:4)	C_18_H_24_O_5_	Zearalanone, β-,α-Zearalenol	Nonsteroidal estrogen	Mycotoxin It has major effects on reproduction in females, it causes cytotoxicity, neurotoxicity, and oxidative stress at the molecular level	[119,120]

**Table 4:** List of most abundant polyphenols shared by the two algal species *Asparagopsis armata* and *Rugulopteryx okamurae*. Possible top isomers and their associated identified bioactivities are attached.

Formulas	Top Isomer(s)	Bio-Activities	References
C_6_H_6_O_3_	Maltol	An antioxidant found in Korean red ginseng	[85]
	Pyrogallol	Highly cytotoxic effect on human lung cancer cell lines, induces apoptosis in endothelial cells. Antibacterial activity against *Staphylococcus epidermidis* and *Staphylococcus aureus*	[84,87,88]
C_8_H_8_O_2_	Phenylacetic acid	Antimetabolite useful in cancer chemotherapy	[121]
C_6_H_6_O_4_	Kolic acid	NF-kappaB inhibitor, skin lightening agent, an EC 1.10.3.1 (catechol oxidase), EC 1.10.3.2 (laccase), EC 1.13.11.24 (quercetin 2,3-dioxygenase), EC 1.14.18.1 (tyrosinase), and EC 1.4.3.3 (D-amino-acid oxidase) inhibitor	[122]
C_10_H_10_O_4_	Ferulic acid	Antioxidant, a MALDI matrix material, a plant metabolite, an anti-inflammatory agent, an apoptosis inhibitor, and a cardioprotective agent	[123]
C_11_H_10_O_4_	Scoparone	Major constituent of the Chinese herbal medicine Yin Chen Hao, exhibits anti-inflammatory, anti-allergic, and anti-tumor activities	[124]
C_10_H_8_O_5_	Fraxetin	An *Arabidopsis thaliana* metabolite, antimicrobial agent, apoptosis inhibitor and inducer, antioxidant, anti-inflammatory, hepatoprotective agent, antibacterial, and hypoglycemic agent	[125]
C_12_H_12_O_4_	Hispolon	Anti-cancer activity	[83]
	Eugenitin	Anti-leishmanial and cytotoxicity assays	[89]
C_14_H_12_O_3_	Resveratrol	A phytoalexin, antioxidant, glioma-associated oncogene inhibitor, and geroprotector found in high concentrations in red grapes	[86]
C_14_H_14_O_4_	Marmesin	Potential natural UV-A-filtering product	[90]

### 2.3. Antioxidant Capacity

**Figure 4:**
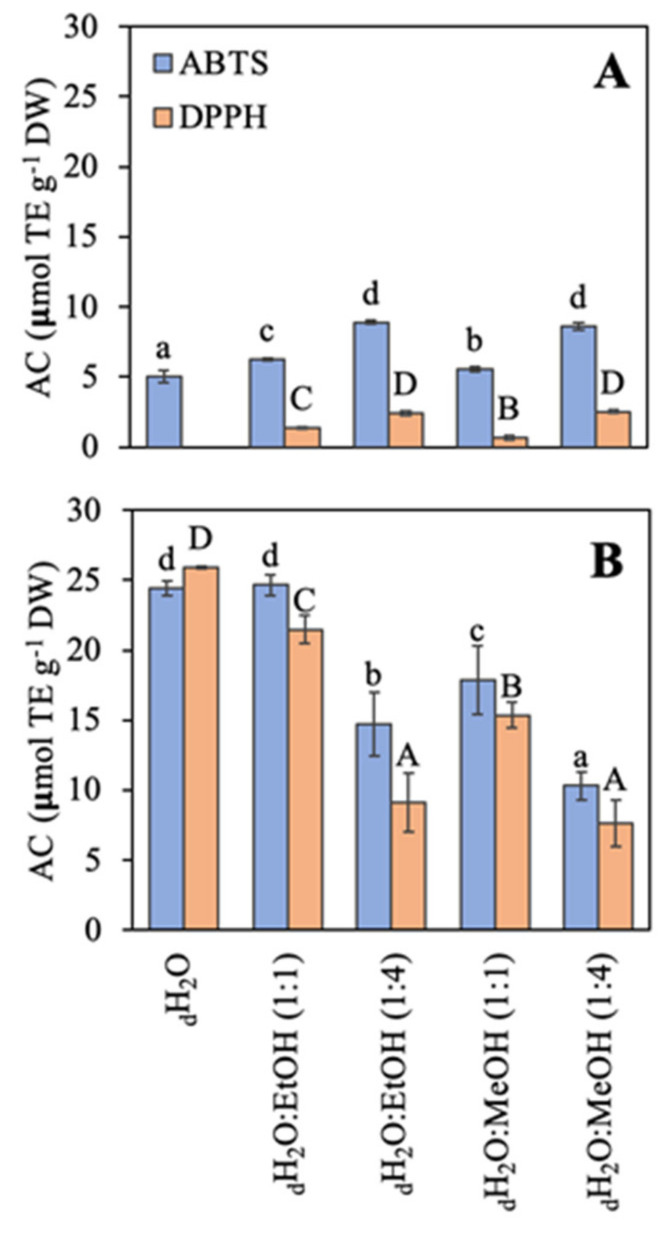
Antioxidant capacity (AC) using two methodologies expressed as μmol TE g^−1^ DW of the different extracts in different solvents of water (_d_H_2_O), _d_H_2_O:Ethanol (EtOH) (1:1), _d_H_2_O:EtOH (1:4), _d_H_2_O:Methanol (MeOH) (1:1), and _d_H_2_O:MeOH (1:4), from the biomass of (**A**) *Asparagopsis armata* and (**B**) *Rugulopteryx okamurae*. Values are expressed as average ± standard deviation (SD) (*n* = 3). Different letters indicate significant differences among solvents for each species (ANOVA, *p* < 0.05, SNK test).

The antioxidant capacity (AC) using two different methodologies (ABTS and DPPH) based on the free radical scavenging activity is shown (Figure 4). In general, the antioxidant capacity was higher in *R. okamurae* than in *A. armata*. The AC of *R. okamurae* was similar with both methodologies (only in three cases significant slight differences were observed), and with both methodologies, the AC was highest in the most polar solvents: _d_H_2_O ≥ _d_H_2_O:EtOH (1:1) > _d_H_2_O:MeOH (1:1) > _d_H_2_O:EtOH (1:4) ≥ _d_H_2_O:MeOH (1:4). In contrast, *A. armata* presented the highest AC using the ABTS method. Values obtained with the DPPH assay were very low, and when water was used as solvent no signal was detected.

### 2.4. Antimicrobial Activity 

The antimicrobial activity of the different extracts is shown in Table 5. Concerning human pathogens, *R. okamurae* inhibited the bacterial growth of *S. aureus* and *C. acnes* in both _d_H_2_O:EtOH (1:4) and _d_H_2_O:MeOH (1:4) extracts at a concentration of 50 and 25 mg mL^−1^, respectively. *A. armata* inhibited the growth of *S. aureus* and *P. aeruginosa* in both _d_H_2_O:EtOH (1:4) and _d_H_2_O:MeOH (1:4) extracts at a concentration of 25 and 50 mg mL^−1^, respectively. Aqueous extracts did not inhibit any of the human pathogens. In the case of fish pathogens, none of the extracts of *R. okamurae* inhibited any bacteria. However, different extracts of *A. armata* inhibited bacterial growth. The minimum concentration to inhibit *V. anguillarum* was 6.25 mg mL^−1^ in the case of _d_H_2_O:EtOH (1:4). *V. harveyi* and *T. maritimum* were only inhibited by aqueous extract at a concentration of 50 and 25 mg mL^−1^, respectively. *P. damselae* subsp. *piscicida* was inhibited by both _d_H_2_O:EtOH (1:4) and _d_H_2_O:MeOH (1:4) at a concentration of 50 mg mL^−1^. *T. soleae* and *T. gallaecium* were inhibited by all the extracts tested at a concentration of 12.5 mg mL^−1^. Finally, *A. hydrophila* was also inhibited by all the extracts tested at a concentration of 25 mg mL^−1^.

### 2.5. Correlations and Principal Component Analysis

The Pearson correlation (Table S2) analysis showed a positive correlation (*p* < 0.01) between the total phenolic content (without and with PVPP) and both antioxidant capacity methodologies. Phenols content also showed a positive correlation with CHO, CHOP, and highly saturated O poor molecules. However, a negative correlation was obtained with CHON, CHOS, aromatic O rich, highly saturated O rich, unsaturated O rich, unsaturated N, and saturated O poor compounds. Antioxidant capacity (ABTS and DPPH) showed a positive correlation with CHO and highly saturated O poor (only ABTS), and a negative correlation with CHON, CHOS (only ABTS), aromatic O rich (only ABTS), unsaturated O rich, unsaturated N, and saturated O poor compounds.

**Figure 5:**
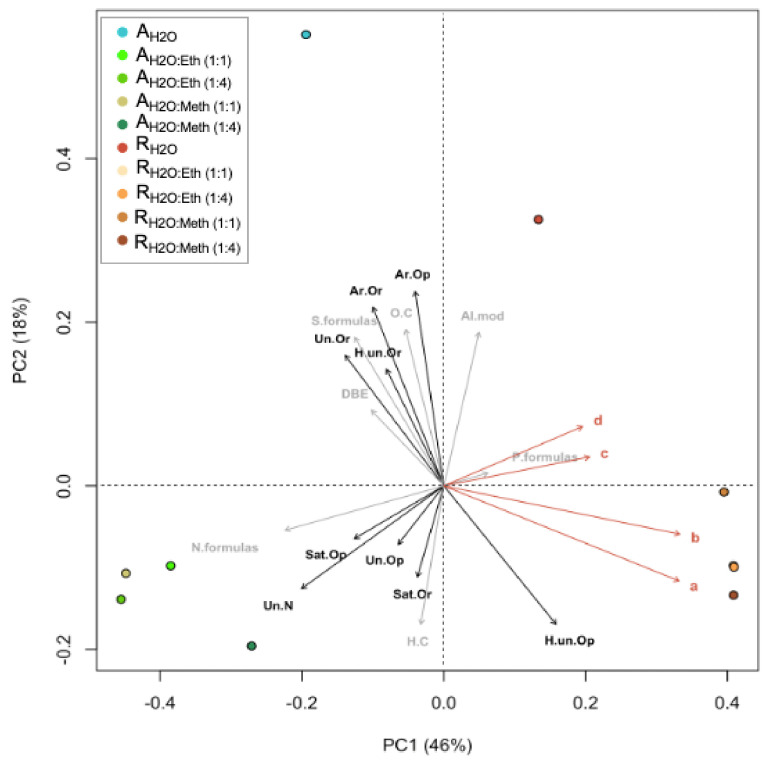
Principal coordinate analyses (PCoA) based on Bray–Curtis dissimilarities of the relative abundance of molecular formulas in *Asparagopsis armata* and *Rugulopteryx okamurae*. The percentages give the molecular variability as explained by the axes. Chemical/bioactivity parameters are indicated by orange arrows: (**a**) phenolic compounds (no PVPP) (mg g^−1^ DW), (**b**) phenolic compounds (PVPP) (mg g^−1^ DW), (**c**) antioxidant capacity (ABTS) (μmol TE g^−1^ DW), and (**d**) antioxidant activity (DPPH) (μmol TE g^−1^ DW). Molecular parameters are indicated by gray arrows: formulas with N, S, and P content (N. formulas, S. formulas, P. formulas), O/Cw, H/Cw, and DBEw. Molecular categories are indicated by black arrows: aromatic (Ar), highly unsaturated (H. un), saturated (Sat), unsaturated (Un), oxygen rich (Or), oxygen poor (Op). All parameters were fitted onto the ordination. All correlations with chemical/bioactivity parameters were significant (*p* < 0.001). The projections of sampling points onto the vector arrows show maximum correlations with the corresponding chemical/bioactivity parameters, molecular parameters, and molecular categories.

In PCoA analysis, samples were correlated to nine chemical/bioactive parameters (Figure 5). In the ordination plots, the first two axes explained 64% of the algal extract’s molecular variability. In PCoA, the projection of sample points onto the vectors depicts correlations with the corresponding molecular/bioactivity parameters. PCoA and cluster analyses revealed two molecular clusters in relation to the two species evaluated and two independent samples, which correspond to the _d_H_2_O extracts. The *R. okamurae* cluster correlated strongly with phenolic compounds (both no PVPP and PVPP) and antioxidant capacity (both ABTS and DPPH) (Figure 5). *R. okamurae* extracts correlated with highly unsaturated (O poor) molecules. *A. armata* extracts correlated with C/H_w_, N formulas, unsaturated (O poor and with N), and saturated (O rich and O poor). The *R. okamurae* _d_H_2_O extract correlated significantly with AImod_w_, whereas the *A. armata* _d_H_2_O extract correlated with S-enriched formulas, O/C_w_ ratio, DBE_w_, aromatics (O rich and O poor), highly unsaturated (O rich) and unsaturated (O rich).

## 3. Discussion

Marine organisms present a wide biochemical composition with promising applications, although thousands of molecules remain still uncovered [126,127,128,129,130]. Non-target metabolomic studies are required to discover the role of new molecules, which aim to examine all detectable metabolites (specific to isolation and analytical method) [131]. This approach has the power to identify relevant and new metabolites at a faster pace with the appropriate data analysis tools [132]. High-resolution mass spectrometry (HR-MS), coupled with bioinformatics tools, has gained momentum in this type of study [133,134]. Ultrahigh-resolution FT-ICR-MS can address the molecular diversity of a sample most closely and allows a first molecular screening, noting that some compound classes escape MS detection as they do not ionize efficiently. Because of the high mass accuracy and resolution, this method detects thousands of molecular formulas in complex mixtures without iterative fractionation [135,136]. However, HR-MS is unable to differentiate between structural isomers of a molecular formula [137,138].

In the first instance, the internal composition of both invasive species (*Asparagospsis armata* and *Rugulopteryx okamurae*) was analyzed through elemental analysis and spectrophotometric methods. In general, *A. armata* presented the highest content of nitrogenous compounds (proteins) and minerals (ash), whereas *R. okamurae* was rich in carbonated compounds (total carbon or lipids). Compared to other studies, Regal et al. [139] observed similar content in the ash (~45%) or lipids (~3%) content in *Asparagopsis taxiformis*. On the other hand, this study showed a lower protein content than that observed by other authors (11 vs. 19%, respectively) [139,140]. Jard et al. [141] also observed a high sulphur content in comparison to other species. The results obtained for *R. okamurae* were in line with the review by Bogaert et al. [142] in the genus *Dyctiota*: inorganic elements (or ashes) varied from 17–30%, total carbohydrates varied from 10–54%, or total proteins varied from 1.7–27.6%, among others. High content of lipids seems to be common in this genus (0.5–20.2%). These results are in accordance with the heteroatomic composition obtained after the FT-ICR-MS, in which the highest content of CHON and CHOS formulas were observed in *A. armata*. Marked differences in the internal composition were observed between species.

Phenolic compounds were quantified in the different extracts. These compounds are mainly present in brown algae, although some red and green algae can also have relevant content of phenols [143,144]. In this work, *A. armata* presented a higher or similar phenol concentration compared to that obtained by other authors [139,140,143]. The content of phenolic compounds found in *R. okamurae* were in the range of other brown seaweeds, although it did not reach the highest values observed in *Sargassum* sp. or *Cystoseira* sp. [37,145,146,147,148]. After the treatment with PVPP, the phenol content drastically decreased in both species (mainly in *A. armata*). These results indicate a possible interference with other compounds (e.g., aromatic amino acids, sugars, ascorbic acid, or sulfite, among others) in the methodology [149]. Other authors obtained a lower decrease in the phenols content in different brown algae such as *Cystoseira, Sargassum, Fucus,* or *Ascophyllum* [144,150] species that present a higher phenol content than *R. okamurae*. There is little information about the use of PVPP in red algae extract. Vega et al. [144], analyzed the phenolic content before and after the use of PVPP in some red algae (*Pterocladiella capillaceae*, *Hypnea spinella*, *Dermocorynus dichotomus,* and *Halopithys incurva*), and after the use of PVPP, phenols were only detected in *H. incurva*.

A first molecular screening of these invasive species has been performed in this work by using FT-ICR-MS, and the presence of different bio-compounds has been suggested. To facilitate the search for published bioactivities and possible applications in the founded molecules, the software *ChemCrawler* was used. *ChemCrawler* will facilitate the selection of those samples with an apparent higher potential and will direct the next steps to be followed for the detection and isolation of the molecule of interest. 

Metabolomic information obtained from marine algae is still very limited in comparison to terrestrial plants. Most of the studies done with algae extracts were focused on the identification/quantification of selected compounds (named as target metabolomics) such as carotenoids, phycobiliproteins, mycosporine-like amino acids, polysaccharides, or fatty acids. Kumar et al. [151] reviewed some metabolic studies in marine macrophytes, identifying compounds involved in chemical defence or adaptative strategies. For example, in *A. armata*, bromoform and dibromoacetic acid with anti-bacterial properties were identified [41]. In *Dyctiota* sp., some terpenoids such as pachydictyol A, dictyol B/E/H, or 10–18- diacetoxy-8-hydroxy-2,6-dolabelladiene were identified [152]. Non-targeted metabolic studies in seaweed have identified differences in the metabolome profile of brown, green, and red species [153], or have observed variabilities in the metabolome among species from the same genus [154]. Nylund et al. [155] studied the chemical defence responses of the invasive species *Gracilaria vermiculophylla* using mass spectrometry techniques in combination with bioassays. More recently, Dixit et al. [156] identified 16 potential metabolites in *Dictyota dichotoma* using liquid chromatography with time-of-flight mass spectrometry. 

Among the most abundant molecules found in this work, some of them have previously been observed. Gallic acid, found in the most abundant molecules in *A. armata*, is one of the most commonly reported phenols in green, red, and brown seaweeds [157,158]. In *R. okamurae*, three different prostaglandins were identified. This type of molecule was also identified by Nylund et al. [155] in the invasive red algae *G. vermiculophyla*. Dixit et al. also observed coumarins and different terpene lactones in *Dictyota dichotoma* [156].

A deeper search was done for common phenolic compounds and terpenoids. These molecules can be involved in the chemical defence of the algae and, for instance, in the success of their invasion. In addition, they are also well known for their possible applications in the industry. Phenolic compounds can have wide applicability such as antidiabetic, antiobesity, cardiometabolic, neuroprotective, UV protective, anticancer, and antimicrobial activity, most of them related to their antioxidant activity [157]. Terpenoids have also been described to have a wide range of applications, such as anti-herbivory, anti-inflammatory, anti-fouling, anti-tumoral, or insecticidal properties [48,50,51,152].

There is limited information about the phenolic compounds profile in seaweeds. Pinto et al. [159] observed that polar extracts of *A. armata* were rich in bromiated phenolics such as caffeic or p-coumaric acid. Zhong et al. [160] analyzed the phenolic profile of 8 different seaweeds and identified a total of 54 compounds. Recently, different diterpenoids have been identified in *R. okamurae* [50,51]. One of them, dilkamural, was observed in a higher amount in *R. okamurae* and tracked down in sea urchins (*Paracentrotus lividus*) fed with these algae, observing not only deterrent properties but also harmful and even lethal effects over the sea urchins [51]. In this work, one of the more abundant diterpenoids (C_20_H_32_O) can be associated with Pachydictyol A [152]. 

Two different bioactivities were analyzed in vitro. The antioxidant capacity can protect the cell against oxidative stress, which have been described to be involved in different pathologies such as certain cancers, arthritis, Parkinson´s disease, gastrointestinal diseases, or aging [161,162,163,164]. Several authors have studied the antioxidant capacity of *A. armata* using different methodologies (i.e., DPPH, ORAC, β-carotene linoleic acid system, reducing activity, nitrite oxide radicals, copper chelating activity, or ion chelating activity). Pinteus et al. [35] and Felix et al. [165] reviewed the antioxidant capacities observed in *A. armata*, among other possible biotechnological applications. Different expression units have been used by the authors, so it is difficult to be compared with the results presented in this work. Most of the results have been obtained using organic solvents. In this work, the antioxidant capacity of aqueous extracts has been studied, showing even higher antioxidant capacity in comparison to the organic solvents. In *R. okamurae*, very little information on antioxidant capacity has been published. Brown algae normally present higher antioxidant capacity values than red or green algae [143,144], although *R. okamurae* did not show such a high antioxidant capacity [148]. Schneider et al. [148] observed even lower values than those obtained in this work. Some authors have studied the antioxidant capacity of other species from the Dyctiotaceae family (*Dictyota* sp. or *Padina* sp.) [166,167,168], although the different expression units make difficult the comparisons.

The antimicrobial activity of *A. armata* and *R. okamurae* extracts was evaluated against six human bacteria. *R. okamurae* inhibited two opportunistic bacteria related to skin infection, *Staphylococcus aureus* and *Cutibacterium acnes*. The second one, *C. acnes*, is the bacteria responsible for acne vulgaris [169]. *A. armata* also showed the capacity to inhibit *S. aureus* and *P. aeruginosa*. Other authors also observed antimicrobial activity against *S. aureus* and *P. aeruginosa* in species such as *Sargassum wightii*, *Padina tetrastromatica*, *Ulva fasciata,* or *Hypnea pannosa* [170,171]. These authors obtained the highest values using organic solvents such as dyethilether or methanol:toluene (3:1) in fresh biomass. Lee et al. [172] analyzed the capacity to inhibit *C. acnes* of several seaweeds from Korea and observed that ethanolic extract of *R. okamurae* could inhibit its growth with an MIC of 256 µg mL^−1^. Other ethanolic and methanolic plant extracts exhibited inhibitory activity against *C. acnes* [173,174], ranging from 7.81 to 32 µg mL^−1^. Regarding fish pathogens, while *R. okamurae* did not inhibit any of the strains assayed, *A. armata* inhibited several fish pathogens. The increasing bacterial resistance against commercial antibiotics has led to the search of new sources of antimicrobial compounds. Marine algae are promising sources of new antibacterial compounds. In aquaculture, the resistance against antibiotic is causing a significant economic loss in the industry associated with mortalities [175,176,177,178]. The results suggest the possible use of *R. okamurae* extracts in skin care products as natural anti-acne or antibacterial compounds, and that *A. armata* extracts could be a promising, safe, and environmentally friendly alternative to antibiotics in the treatment of fish diseases, which could have a tremendous impact on the aquaculture sector. Nonetheless, the extraction method and solvent polarity could modify the antimicrobial potential of the algae biomass, i.e., the use of novel extraction techniques could increase its activity [177]. Future studies determining the toxicity and optimal doses in in vivo trials are necessary to confirm our positive in vitro results. 

Other bloom producers of brown algae, autochthonous or invaders, have been analyzed for their possible valorization and exploitation. For example, *Sargassum* sp. has been studied for its possible use in bioremediation, bioenergy production, or obtention of target molecules such as alginates or fucoidans with interest in the pharmaceutical industry [18,178,179,180,181]. In relation to the genus *Asparagopsis*, different authors have analyzed possible applications for its valorization [35,165,182]. 

Recently, the drastic colonization of *R. okamurae* in the Strait of Gibraltar has been related to the increase in temperature and nutrients in the water column [28,183]. Just in the last four years, millions of tonnes per year of *R. okamurae* biomass have been collected on the Andalusian coast as beach-cast algae. On this basis, a high amount of biomass is available for the extraction of interesting bio-compounds that could help to control the invasive species. FT-ICR MS cannot give us precise information on enzymes or molecules related to the acclimation to physical and chemical variables, but, on the other hand, information about molecules related to the success of invasion as anti-herbivory, antibacterial, and antiviral activities, among others, could be obtained. 

## 4. Materials and Methods

### 4.1. Algal Material

Two invasive species in southern Spain were used in the study. *Asparagopsis armata* was collected in Punta Chullera (Manilva, Malaga; 36°18′38.8″ N, 5°14′55.8″ W) in June 2019, and *Rugulopteryx okamurae* was collected in Playa de la Caleta (Tarifa, Cadiz; 36°00′41.7″ N, 5°35′59.3″ W) in May 2019. Algae biomass was transported to the laboratory in a portable fridge at 4 °C, washed and cleaned to remove salt, sand, and epiphytes, frozen at −40 °C, and freeze-dried (Cryodos, Telstar, Barcelona, Spain).

### 4.2. Preparation of Algal Extracts

Lyophilized samples were ground with a coffee grinder to a powder and extracted in distilled water (_d_H_2_O) and different combinations of ethanol (EtOH) and methanol (MeOH) in _d_H_2_O, _d_H_2_O:EtOH (1:1), _d_H_2_O:EtOH (1:4), _d_H_2_O:MeOH (1:1), and _d_H_2_O:MeOH (1:4). Two and a half grams of dry biomass was homogenized using Ultra-Turrax (T-25 digital, IKA, Germany) in 50 mL of the different solvents. Extracts were incubated at 45 °C for 2 h in a water bath. Extracts were filtered and centrifuged (Labofuge 400R, Heraeus, Kendro Laboratory products, Langenselbold, Germany). For the molecular analysis, extracts were dried under vacuum in a rotary evaporator (Jouan RC 10-09, France).

### 4.3. Biochemical Composition 

#### 4.3.1. Internal Carbon, Nitrogen, and Sulphur

Total internal carbon, nitrogen, and sulphur contents were determined from dry biomass of algae using an elemental analyzer (CNHS-932, LECO, St. Joseph, MI, USA) in the Research Support Central Services of the University of Malaga. 

#### 4.3.2. Ashes

Ashes were determined by combustion of the dry biomass at 500 °C for 12 h in a muffle oven. The organic matter was estimated by measuring the weight loss before and after the mentioned combustion.

#### 4.3.3. Total Proteins

Total proteins were calculated using the nitrogen-to-protein conversion factor of 4.92 for seaweeds [184]. 

#### 4.3.4. Carbohydrates

Carbohydrates were quantified according to the phenol-sulfuric acid method by DuBois et al. [185]. A total of 5 mg of dry biomass was homogenized in 5 mL of 1 M H_2_SO_4_ and incubated for 1 h at 100 °C. For the reaction, 1 mL of the extract was mixed with 1 mL of 5% phenol and 5 mL of concentrated H_2_SO_4_. Absorbance was measured at 485 nm (UV-mini-1249, Shimadzu, Kyoto, Japan). Glucose was used as a standard.

#### 4.3.5. Lipids

Lipids were determined using the gravimetric method described by Folch et al. [186]. A total of 200 mg of dry biomass was homogenized in 5 mL of chloroform:methanol (2:1). The lipid fraction was separated by adding 2 mL of KCl 0.88% and by centrifugation. Lipids were quantified gravimetrically once the solvent (chloroform) was completely evaporated with nitrogen.

#### 4.3.6. Phenolic Compounds

Total phenolic compounds (TPC) were determined from the different extracts using the Folin–Ciocalteu method according to Singleton & Rossi [187] with some modifications. A total of 100 μL of the extracts obtained in item 2.3 were mixed with 700 μL of distilled water and 50 μL of Folin–Ciocalteu phenol reagent (Sigma-Aldrich, St. Louis, MO, USA). After the vortex, 150 μL of Na_2_CO_3_ 20% was added and samples were mixed again. The mixtures were incubated for 2 h at 4 °C in darkness. Absorbances were measured at 760 nm. Phloroglucinol was used as standard. Treatment of the extracts with polivinilpolipirroline (PVPP) was also used to eliminate possible interference with proteins or amino acids in the method [149]. PVPP is an insoluble polymer that can bind small phenolic compounds such as phlorotannins (van Alstyne, 1995) [188]. The difference between the measurements with and without PVPP can provide a more accurate phenols content.

### 4.4. Molecular Diversity and Published Bioactivities

The molecular diversity was determined using Fourier-transform ion cyclotron resonance mass spectrometry (FT-ICR-MS). A total of 1 mL of the different algae extracts was dried and diluted with ultrapure H_2_O:MeOH (1:1). Dilutions at a concentration of 2.5 mg C L^−1^ were prepared. Duplicates of each extract were measured using a Solarix FT-ICR-MS (Bruker Daltonik GmbH) connected to a 15 Tesla superconducting magnet (Bruker Biospin). Samples were injected at a flow rate of 120 µL h^−1^ into the electrospray ionization source in negative mode. Ions were accumulated in the hexapole for 0.1 s prior to transfer into the ICR cell. Samples were acquired from 100 to 1000 Da with an accumulation of 200 scans. The calibration of the spectra resulted in a mass error of <0.1 ppm. Instrument assessment was done with an in-house molecularly stable standard [189,190]. 

The software ICBM-OCEAN was used to eliminate instrumental noise (i.e., method detection limit (MDL) of 2.5), mass alignment, and molecular formula attribution [191]. In the formula attribution, the N, S, P rule, isotope verification, and homologous series were applied to exclude unlike formulas and improve formula assignment [192]. Identified contaminants were removed before statistical analyses. Only the formulas present in duplicated measurements were considered for further analyses. 

The obtained formulas were sorted into groups containing the atoms CHO, CHON, CHOS, CHOP, CHONS, CHOSP, and CHONP (the latter three are referred to as “others”). The identified molecular formulas were distributed into compound groups, namely (1) aromatics (AImod > 0.5), (2) polyphenols (0.5 ≤ Aimod ≤ 0.666), (3) highly unsaturated (Aimod < 0.5, H/C < 1.5), (4) unsaturated (1.5 ≤ H/C ≤ 2), and (5) saturated (DBE = 0). The five molecular categories were subdivided into oxygen rich (O rich, O/C > 0.5) and oxygen poor (O poor, O/C ≤ 0.5), with an extra category for the unsaturated category named “with N” (1.5 ≤ H/C ≤ & N) [191]. The formulas were normalized to the sum of all molecular formula intensities for each sample, and, subsequently, the intensity weighted averages (indicated by the subscript w) of elemental ratios H/C_w_ and O/C_w_, DBE_w_, and AImod_w_ were calculated [193].

*ChemCrawler* was introduced in this work as a novel software-based tool for finding relevant information about chemical compounds. Developed within the context of this research project in collaboration with ValueData GmbH, *ChemCrawler* utilizes publicly available data to provide information on the most common isomers for a given molecular formula. In addition, it provides a selection of abstracts of relevant publications related to these isomers, enabling fast and convenient analysis of their potential pharmaceutical and biotechnological applications. *ChemCrawler* is a web application developed using the Python programming language and open-source libraries such as *Flask* [194] and *requests* [195]. To initiate a search, the user enters a molecular formula (Figure S1), which is then fed into an algorithm systematically searching for relevant data in the open-access databases *PubChem* [196] and *MetaboLights* [197]. Programmatic access to PubChem is implemented using its representational state transfer (REST) interface, which allows complex queries on chemical and bibliographic data [198]. Entries in the MetaboLights database are queried in a similar manner [199]. Once all relevant information has been collected, a concise report consisting of each isomer and its respective references is generated and made available via the web interface (Figure S1). By clicking on one of the references provided, the user is redirected to the original data source for further information. At present, *ChemCrawler* is available for internal use only, however, access to this service may be expanded upon further interest in the scientific community.

### 4.5. Antioxidant Capacity (ABTS and DPPH) 

Antioxidant capacity (AC) of the different extracts (obtained in item 2.3) was evaluated through two different methods based on the free radical scavenging activity:

The ABTS assay was performed according to Ree et al. [200] with some modifications. The ABTS radical cation (ABTS^+•^) was generated by a reaction of 7 mM ABTS (2,2-azino-bis(3- ethylbenzothiazoline-6-sulfonic acid)) and 2.45 mM K_2_S_2_O_8_ (potassium persulphate) in phosphate buffer solution (PBS: 0.1M; pH: 6.5). This reaction was stored for 12-16 hours at room temperature to ensure the complete formation of the radical. ABTS^+•^ solution was diluted with PBS until the absorbance at 727 nm was around 0.75 ± 0.05. For the reaction, 50 μL of the samples was mixed with 950 μL of the diluted ABTS^+•^. The mixture was incubated for 8 minutes at room temperature and darkness, and absorbance was measured at 727 nm. 

The DPPH assay was made according to Brand-Williams et al. [201] with some modifications. For the reaction, 200 μL of the samples was mixed with 1 mL of the DPPH (2,2-diphenyl-1-picrylhydrazyl) solution (0.06 mM of DPPH in methanol 80%). After 30 minutes of incubation at room temperature and darkness, absorbance was measured at 517 nm.

For both methods, a standard solution of Trolox (6-hydroxy-2,5,7,8-tetramethylchroman-2-carboxylic acid) was used as a reference, and the results were expressed as μmol TE (Trolox equivalent) g^−1^ DW.

### 4.6. Antimicrobial Activity

The antimicrobial activity of the _d_H_2_O, _d_H_2_O:EtOH (1:4), and _d_H_2_O:MeOH (1:4) extracts were analyzed for different human and fish pathogens:

Human pathogens: *Cutibacterium acnes* (CECT 5684, obtained from the Spanish Culture Collection) was cultivated in Reinforced Clostridial Medium (RCM) (#CM0149, Oxoid Ltd., Basingstoke, Hampshire, England) supplemented with 1.5% bacteriological agar (RCMA) (#LP0011, Oxoid). As this strain is anaerobic, the plates were incubated under anoxic conditions at 37 °C for 24-48 h using a GasPak anaerobic system (BD GasPak^TM^ EZ Container System, Becton Dickinson & Co., Sparks, BD, USA). *Escherichia coli*, *Pseudomonas aeruginosa*, *Salmonella enterica,* and *Staphylococcus aureus* (obtained from the stock collection of the Microbiology Department of the University of Málaga) were cultivated in Tryptic Soy Agar (TSA, Oxoid) for 24 h at 37 °C.

Fish pathogens: *Tenacibaculum maritimum, T. soleae,* and *T. gallaecium* (obtained from the stock collection of the Microbiology Department of the University of Málaga) were cultivated in *Flexibacter maritimum* medium (FMM; Condalab, Madrid, Spain) for 48 h at 28 °C (FMMA). *Vibrio anguillarum* (CECT 522, obtained from the Spanish Culture Collection), *Vibrio harveyi* Lg16/00, *Photobacterium damselae* subsp. *piscicida* Lg41/01, and *Aeromonas hydrophila* Lg28/4 (strains isolated from diseased specimens of *Solea senegalensis* and stocked by the Microbiology Department of the University of Malaga) were cultivated in TSA supplemented with 1.5% NaCl (TSAs) for 24–48 h at 22 °C.

Firstly, serial dilutions of the different algae extracts were made. The antimicrobial activity was tested using the diffusion method [202] with some modifications. In brief, bacterial colonies were suspended in saline solution and adjusted to OD_600_ = 0.2 (10^8^ cfu mL^−1^, approximately). The bacterial suspension was spread over the surface of RCMA, FMMA, TSA, and TSAs agar plates (5 mm agar thickness) to obtain an even inoculum. For aqueous extracts, wells of 6 mm diameter were made in the agar and then filled with different algae extract concentrations. _d_H_2_O was added as a negative control. For hydroalcoholic extracts, a sterilized filter paper disc of 6 mm diameter (Whatman no. 1 filter paper) was used. An inoculum of 10 μL for each extract concentration was transferred to each disc. As a negative control, different solvents were used. The disks were dried at 37 °C for 15 min to evaporate the ethanol and methanol, and then the discs were placed over the plates with the bacteria. Plates were incubated for 24–48 h (depending on the bacteria). The antimicrobial activity was determined by the presence/absence of an inhibition area around each well or disc. The lowest extract concentration that inhibited bacterial growth was designated as the minimum inhibitory concentration (MIC).

### 4.7. Statistical Analysis

The software STATISTICA (V.7) was used for statistical analysis. Student’s t-test and analysis of variance (ANOVA) were used to compare the obtained data. Student’s t-test was performed to determine significant differences between species. One-way ANOVA was used to evaluate the effect of the solvents in the different analyses (TPC and AA). A Student–Newman–Keuls (SNK) post hoc test was performed to obtain significant groups after significant interaction in the ANOVA. Homogeneities of variance was tested using the Cochran test and by visual inspection of the residuals. Correlations among variables were calculated using Pearson´s correlation coefficients.

Principal coordinate analysis (PCoA) was performed on a Bray–Curtis dissimilarity matrix of the normalized peak intensities of all identified molecular formulas. For the analyses, extracts were included in triplicates. The molecular categories, phenolic compounds (with and without PVPP), and antioxidant capacity (ABTS and DPPH) were fitted post hoc to the PCoA scores using the envfit function of the vegan package [203]. The correlation of chemical/bioactivity parameters to the molecular composition (PCoA) was tested with 10,000 permutations.

## Figures and Tables

**Table 1:** Content of organic matter, ashes, total internal carbon, nitrogen and sulphur, total proteins, carbohydrates, lipids (%), and C:N ratio from the biomass of *Asparagopsis armata* and *Rugulopteryx okamurae*. Values are expressed as average ± standard deviation (SD) (*n* = 3).

	*A. armata*	*R. okamurae*
Organic matter	58.2 ± 1.4	72.2 ± 1.4
Ashes	41.8 ± 1.4	27.8 ± 1.4
Carbon	19.0 ± 0.9	35.3 ± 0.3
Nitrogen	2.3 ± 0.3	1.7 ± 0.1
C:N	8.6 ± 0.7	19.9 ± 1.0
Sulphur	1.9 ± 0.3	0.8 ± 0.1
Total proteins	10.56 ± 0.8	9.15 ± 0.6
Carbohydrates	13.73 ± 2.1	8.8 ± 0.4
Lipids	2.5 ± 0.1	8.0 ± 0.4

**Table 5:** Antimicrobial activity of the aqueous (_d_H_2_O), ethanolic (_d_H_2_O:Ethanol (EtOH) (1:4)), and methanolic (_d_H_2_O:Methanol (MeOH) (1:4)) extracts from the biomass of *Asparagopsis armata* and *Rugulopteryx okamurae*. The results are expressed as the minimum inhibitory concentration (MIC, mg mL^−1^).

	*R. okamurae*			*A. armata*		
	_d_H_2_O	_d_H_2_O:EtOH (1:4)	_d_H_2_O:MeOH (1:4)	_d_H_2_O	_d_H_2_O:EtOH (1:4)	_d_H_2_O:MeOH (1:4)
**HUMAN**						
*S. aureus*	-	25	25	-	25	25
*E. coli*	-	-	-	-	-	-
*P. aeruginosa*	-	-	-	-	50	50
*S. enterica*	-	-	-	-	-	-
*E. faecium*	-	-	-	-	-	-
*C. acnes*	-	50	50	-	-	-
**FISH**						
*V. anguillarum*	-	-	-	25	6.25	12.5
*V. harveyi*	-	-	-	50	-	-
*P. damselae* subsp. *piscicida*	-	-	-	-	50	50
*T. maritimum*	-	-	-	25	-	-
*T. soleae*	-	-	-	12.5	12.5	12.5
*T. gallaecicum*	-	-	-	12.5	12.5	12.5
*A. hydrophila*	-	-	-	25	25	-

## Supplementary Materials

The following supporting information can be downloaded at: https://www.mdpi.com/article/10.3390/md21010005/s1, Table S1: Molecular composition and presence of molecular groups (percentage with respect to the total of formulas) for the different extractions in different solvents: water (_d_H_2_O), _d_H_2_O:Ethanol (EtOH) (1:1), _d_H_2_O:EtOH (1:4), _d_H_2_O:Methanol (MeOH) (1:1), _d_H_2_O:MeOH (1:4), from the biomass of *Asparagopsis armata* and *Rugulopteryx okamurae*. The average of triplicates for each extract are presented.; Table S2: Pearson coefficient (r) between the different variables analyzed in this work for the different extract of both species (*Asparagopsis armata* and *Rugulopteryx okamurae*). Green: Positive correlation, Red: Negative correlations. **: *p* < 0.01, * *p* < 0.05; Figure S1: ChemCrawler Interface (**a**) and an example of molecular search (**b**)

## References

[1] Schaffelke B., Smith J.E., Hewitt C.L. (2006). Introduced Macroalgae—A Growing Concern. J. Appl. Phycol..

[2] Molnar J.L., Gamboa R.L., Revenga C., Spalding M.D. (2008). Assessing the global threat of invasive species to marine biodiversity. Front. Ecol. Environ..

[3] Raffo M.P., Eyras M.C., Iribarne O.O. (2009). The invasion of *Undaria pinnatifida* to a *Macrocystis pyrifera* kelp in patagonia (Argentina, south-west Atlantic). J. Mar. Biol. Assoc. United Kingd..

[4] Galil B.S., Marchini A., Occhipinti-Ambrogi A. (2018). East is east and West is west? Management of marine bioinvasions in the Mediterranean Sea. Estuar. Coast. Shelf Sci..

[5] Viard F., Comtet T. (2015). Applications of DNA-based methods for the study of biological invasions. Biological Invasions in Changing Ecosystems: Vectors, Ecological Impacts, Management and Predictions.

[6] Sorte C.J.B., Williams S.L., Zerebecki R.A. (2010). Ocean warming increases threat of invasive species in a marine fouling community. Ecology.

[7] Pimentel D., Zuniga R., Morrison D. (2005). Update on the environmental and economic costs associated with alien-invasive species in the United States. Ecol. Econ..

[8] Xu H., Ding H., Li M., Qiang S., Guo J., Han Z., Huang Z., Sun H., He S., Wu H. (2006). The distribution and economic losses of alien species invasion to China. Biol. Invasions.

[9] Vilà M., Basnou C., Pyšek P., Josefsson M., Genovesi P., Gollasch S., Nentwig W., Olenin S., Roques A., Roy D. (2010). How well do we understand the impacts of alien species on ecosystem services? A pan-European, cross-taxa assessment. Front. Ecol. Environ..

[10] Zenetos A., Gofas S., Verlaque M., Cinar M.E., Garcia Raso J.E., Bianchi C.N., Morri C., Azzurro E., Bilecenoglu M., Froglia C. (2010). Alien species in the Mediterranean Sea by 2010. A contribution to the application of European Union’s Marine Strategy Framework Directive (MSFD). Part I. Spatial distribution. Mediterr. Mar. Sci..

[11] Zenetos A., Gofas S., Morri C., Rosso A., Violanti D., Garcia Raso J.E., Cinar M.E., Almogi-Labin A., Ates A.S., Azzurro E. (2012). Alien species in the Mediterranean Sea by 2012. A contribution to the application of European Union’s Marine Strategy Framework Directive (MSFD). Part 2. Introduction trends and pathways. Mediterr. Mar. Sci..

[12] Kraan S. (2017). Undaria marching on; late arrival in the Republic of Ireland. J. Appl. Phycol..

[13] Bunicontro M.P., Marcomini S.C., Casas G.N. (2019). Environmental Impacts of an Alien Kelp Species (*Undaria pinnatifida*, Laminariales) Along the Patagonian Coasts. Coastal Research Library.

[14] Qi L., Hu C., Xing Q., Shang S. (2016). Long-term trend of *Ulva prolifera* blooms in the western Yellow Sea. Harmful Algae.

[15] Cope R.C., Ross J.V., Wittmann T.A., Watts M.J., Cassey P. (2019). Predicting the Risk of Biological Invasions Using Environmental Similarity and Transport Network Connectedness. Risk Anal..

[16] Sardain A., Sardain E., Leung B. (2019). Global forecasts of shipping traffic and biological invasions to 2050. Nat. Sustain..

[17] Zhang J., Shi J., Gao S., Huo Y., Cui J., Shen H., Liu G., He P. (2019). Annual patterns of macroalgal blooms in the Yellow Sea during 2007–2017. PLoS ONE.

[18] Pacheco D., Araújo G.S., Cotas J., Gaspar R., Neto J.M., Pereira L. (2020). Invasive Seaweeds in the Iberian Peninsula: A Contribution for Food Supply. Mar. Drugs.

[19] Ministerio Para la Transición Ecológica y el Reto Demográfico Catálogo Español de Especies Exóticas Invasorasicono Barra Herramientas. https://www.miteco.gob.es/es/biodiversidad/temas/c.

[20] Horridge G.A. (1951). Occurrence of asparagopsis armata harv. on the scilly Isles. Nature.

[21] Streftaris N., Zenetos A. (2006). Alien marine species in the Mediterranean - the 100 ‘worst invasives’ and their impact. Mediterr. Mar. Sci..

[22] Zanolla M., Carmona R., Mata L., De la Rosa J., Sherwood A., Barranco C.N., Muñoz A.R., Altamirano M. (2022). Concise review of the genus Asparagopsis Montagne, 1840. J. Appl. Phycol..

[23] Feldmann J., Feldmann G. (1942). Recherches sur les Bonnemaisoniacées et leur alternance de générations. Ann. Sci. Nat. Bot. Biol. Veg..

[24] El Aamri F., Idhalla M., Tamsouri M.N. (2018). Occurrence of the invasive brown seaweed *Rugulopteryx okamurae* (E.Y. Dawson) I.K. Hwang, W.J. Lee & H.S. Kim (Dictyotales, Phaeophyta) in Morocco (Mediterranean Sea). Mediterr. Fish. Aquac. Res..

[25] Altamirano Jeschke M., De la Rosa Álamos J., Martínez Medina F.J. (2016). Arribazones de la especia exótica *Rugulopteryx okamurae* (E.Y.Dawson) en el Estrecho de Gibraltar. https://riuma.uma.es/xmlui/handle/10630/12433.

[26] Ocaña O., Alfonso-Carrillo J., Ballesteros E. (2016). Massive proliferation of a dictyotalean species (Phaeophyceae, Ochrophyta) through the Strait of Gibraltar (Research note). Rev. la Acad. Canar. Cienc..

[27] García-Gómez J.C., Florido M., Olaya-Ponzone L., Rey Díaz de Rada J., Donázar-Aramendía I., Chacón M., Quintero J.J., Magariño S., Megina C. (2021). Monitoring Extreme Impacts of *Rugulopteryx okamurae* (Dictyotales, Ochrophyta) in El Estrecho Natural Park (Biosphere Reserve). Showing Radical Changes in the Underwater Seascape. Front. Ecol. Evol..

[28] García-Gómez J.C., Sempere-Valverde J., González A.R., Martínez-Chacón M., Olaya-Ponzone L., Sánchez-Moyano E., Ostalé-Valriberas E., Megina C. (2020). From exotic to invasive in record time: The extreme impact of *Rugulopteryx okamurae* (Dictyotales, Ochrophyta) in the strait of Gibraltar. Sci. Total Environ..

[29] Sempere-Valverde J., Ostalé-Valriberas E., Maestre M., González Aranda R., Bazairi H., Espinosa F. (2021). Impacts of the non-indigenous seaweed *Rugulopteryx okamurae* on a Mediterranean coralligenous community (Strait of Gibraltar): The role of long-term monitoring. Ecol. Indic..

[30] García-Gómez J.C., Florido M., Olaya-Ponzone L., Sempere-Valverde J., Megina C. (2021). The Invasive Macroalga *Rugulopteryx okamurae*: Substrata Plasticity and Spatial Colonization Pressure on Resident Macroalgae. Front. Ecol. Evol..

[31] Bermejo R., Vergara J.J., Hernández I. (2012). Application and reassessment of the reduced species list index for macroalgae to assess the ecological status under the Water Framework Directive in the Atlantic coast of Southern Spain. Ecol. Indic..

[32] Bermejo R., Mangialajo L., Vergara J.J., Hernández I. (2014). Comparison of two indices based on macrophyte assemblages to assess the ecological status of coastal waters in the transition between the Atlantic and Mediterranean eco-regions. J. Appl. Phycol..

[33] Figueroa F.L., Flores-Moya A., Vegara J.J., Korbee N., Hernández I. (2012). Autochtonous seaweeds. The Mediterranean Sea: Its History and Present Challenges.

[34] Verlaque M., Steen F., De Clerck O. (2009). Rugulopteryx (Dictyotales, Phaeophyceae), a genus recently introduced to the Mediterranean. Phycologia.

[35] Pinteus S., Lemos M.F.L., Alves C., Neugebauer A., Silva J., Thomas O.P., Botana L.M., Gaspar H., Pedrosa R. (2018). Marine invasive macroalgae: Turning a real threat into a major opportunity - the biotechnological potential of *Sargassum muticum* and *Asparagopsis armata*. Algal Res..

[36] Pereira A.G., Fraga-Corral M., Garcia-Oliveira P., Lourenço-Lopes C., Carpena M., Prieto M.A., Simal-Gandara J. (2021). The Use of Invasive Algae Species as a Source of Secondary Metabolites and Biological Activities: Spain as Case-Study. Mar. Drugs.

[37] Zubia M., Fabre M.S., Kerjean V., Deslandes E. (2009). Antioxidant and cytotoxic activities of some red algae (Rhodophyta) from Brittany coasts (France). Bot. Mar..

[38] Pinteus S., Silva J., Alves C., Horta A., Fino N., Rodrigues A.I., Mendes S., Pedrosa R. (2017). Cytoprotective effect of seaweeds with high antioxidant activity from the Peniche coast (Portugal). Food Chem..

[39] Pinteus S., Rodrigues A., Silva J., Lokman C., Lemos M., Pedrosa R. (2016). The marine invasive *Asparagopsis armata* (Harvey, 1855) as source of bioactive valuable compounds - Antioxidant potential enrichment by Vacuum liquid Chromatography. Front. Mar. Sci..

[40] Pinteus S., Lemos M.F.L., Alves C., Silva J., Pedrosa R. (2021). The marine invasive seaweeds *Asparagopsis armata* and *Sargassum muticum* as targets for greener antifouling solutions. Sci. Total Environ..

[41] Paul N.A., De Nys R., Steinberg P.D. (2006). Chemical defence against bacteria in the red alga Asparagopsis armata: Linking structure with function. Mar. Ecol. Prog. Ser..

[42] Salvador N., Gómez Garreta A., Lavelli L., Ribera M.A. (2007). Antimicrobial activity of Iberian macroalgae. Sci. Mar..

[43] Oumaskour K., Boujaber N., Etahiri S., Assobhei O. (2013). Anti-inflammatory and antimicrobial activities of twenty-tree marine red algae from the coast of Sidi Bouzid (El Jadida-Morocco). Int. J. Pharm. Pharm. Sci..

[44] Rhimou B., Hassane R., Nathalie B. (2010). Antiviral activity of the extracts of Rhodophyceae from Morocco. Afr. J. Biotechnol..

[45] Ochi M., Masui N., Kotsuki H., Miura I., Tokoroyama T. (1982). The structures of fukurinolal and fukurinal, two new diterpenoids from the brown seaweed *Dilophus okamurai* Dawson. Chem. Lett..

[46] Kurata K., Shiraishi K., Takato T., Taniguchi K., Suzuki M. (1988). A New Feeding-Deterrent Diterpenoid from the Brown Alga *Dilophus okamurai* Dawson. Chem. Lett..

[47] Yamase H., Umemoto K., Ooi T., Kusumi T. (1999). Structures and absolute stereochemistry of five new secospatanes and a spatane isolated from the brown alga *Dilophus okamurai* Dawson. Chem. Pharm. Bull..

[48] Suzuki M., Yamada H., Kurata K. (2002). Dictyterpenoids A and B, two novel diterpenoids with feeding-deterrent activity from the brown alga *Dilophus okamurae*. J. Nat. Prod..

[49] De Paula J.C., Vallim M.A., Teixeira V.L. (2011). What are and where are the bioactive terpenoids metabolites from Dictyotaceae (Phaeophyceae). Rev. Bras. Farmacogn..

[50] Cuevas B., Arroba A.I., de los Reyes C., Gómez-Jaramillo L., González-Montelongo M.C., Zubía E. (2021). Diterpenoids from the brown alga *Rugulopteryx okamurae* and their anti-inflammatory activity. Mar. Drugs.

[51] Casal-Porras I., Zubía E., Brun F.G. (2021). Dilkamural: A novel chemical weapon involved in the invasive capacity of the alga *Rugulopteryx okamurae* in the Strait of Gibraltar. Estuar. Coast. Shelf Sci..

[52] Okuyama E., Hasegawa T., Matsushita T., Fujimoto H., Ishibashi M., Yamazaki M. (2001). Analgesic components of Saposhnikovia root (*Saposhnikovia divaricata*). Chem. Pharm. Bull..

[53] National Center for Biotechnology Information PubChem Compound Summary for CID 9858729, Pinellic Acid. https://pubchem.ncbi.nlm.nih.gov/compound/9858729.

[54] Fu J., Zeng Z., Zhang L., Wang Y., Li P. (2020). 4’-o-b-d-glucosyl-5-o-methylvisamminol ameliorates imiquimod-induced psoriasis-like dermatitis and inhibits inflammatory cytokines production by suppressing the nf-kb and mapk signaling pathways. Braz. J. Med. Biol. Res..

[55] Baba K., Tabata Y., Kozawa M., Kimura Y., Arichi S. (1987). Studies on Chinese traditional medicine Fang-feng (I). Structures and physiological activities of polyacetylene compounds from Saposhnikoviae radix. Shoyakugaku Zasshi.

[56] Fuchino H., Murase S., Hishida A., Kawahara N. (2021). Simultaneous UHPLC/MS quantitative analysis and comparison of Saposhnikoviae radix constituents in cultivated, wild and commercial products. J. Nat. Med..

[57] Kreiner J., Pang E., Lenon G.B., Yang A.W.H. (2017). Saposhnikoviae divaricata: A phytochemical, pharmacological, and pharmacokinetic review. Chin. J. Nat. Med..

[58] Meng Y., Yi L., Chen L., Hao J., Li D.X., Xue J., Xu N.Y., Zhang Z.Q. (2019). Purification, structure characterization and antioxidant activity of polysaccharides from *Saposhnikovia divaricata*. Chin. J. Nat. Med..

[59] National Center for Biotechnology Information PubChem Compound Summary for CID 370, Gallic Acid. https://pubchem.ncbi.nlm.nih.gov/compound/Gallic-a.

[60] National Center for Biotechnology Information PubChem Compound Summary for CID 9750, Citrulline. https://pubchem.ncbi.nlm.nih.gov/compound/Citrulli.

[61] Grijalva-Vallejos N., Krogerus K., Nikulin J., Magalhães F., Aranda A., Matallana E., Gibson B. (2021). Potential application of yeasts from Ecuadorian chichas in controlled beer and chicha production. Food Microbiol..

[62] Jang E.Y., Hong K.B., Chang Y.B., Shin J., Jung E.Y., Jo K., Suh H.J. (2020). In Vitro Prebiotic Effects of Malto-Oligosaccharides Containing Water-Soluble Dietary Fiber. Molecules.

[63] National Center for Biotechnology Information PubChem Compound Summary for CID 192826, alpha-Maltotriose. https://pubchem.ncbi.nlm.nih.gov/compound/alpha-Ma.

[64] Lewis S.M., Bucher L., Heitkemper M.M., Harding M., Kwong M., Roberts D. (2016). Medical-Surgical Nursing: Assessment and Management of Clinical Problems.

[65] Perri G., Coda R., Rizzello C.G., Celano G., Ampollini M., Gobbetti M., De Angelis M., Calasso M. (2021). Sourdough fermentation of whole and sprouted lentil flours: In situ formation of dextran and effects on the nutritional, texture and sensory characteristics of white bread. Food Chem..

[66] Sirignano C., Snene A., Rigano D., Tapanelli S., Formisano C., Luciano P., El Mokni R., Hammami S., Tenoh A.R., Habluetzel A. (2017). Angeloylated Germacranolides from Daucus virgatus and Their Plasmodium Transmission Blocking Activity. J. Nat. Prod..

[67] Yan X.H., Chen J., Di Y.T., Fang X., Dong J.H., Sang P., Wang Y.H.U., He H.P., Zhang Z.K., Hao X.J. (2010). Anti-tobacco mosaic virus (TMV) quassinoids from brucea javanlca (L.) merr. J. Agric. Food Chem..

[68] Vineetha R.C., Mathews V.V., Nair R.H. (2021). Ascorbic acid and the mitochondria. Mitochondrial Physiology and Vegetal Molecules: Therapeutic Potential of Natural Compounds on Mitochondrial Health.

[69] National Center for Biotechnology Information PubChem Compound Summary for CID 44208919, PGF2alpha. https://pubchem.ncbi.nlm.nih.gov/compound/prostagl.

[70] National Center for Biotechnology Information PubChem Compound Summary for CID 5280723, Alprostadil. https://pubchem.ncbi.nlm.nih.gov/compound/5280723.

[71] Balaha M., Ahmed N., Geddawy A., Kandeel S. (2021). Fraxetin prevented sodium fluoride-induced chronic pancreatitis in rats: Role of anti-inflammatory, antioxidant, antifibrotic and anti-apoptotic activities. Int. Immunopharmacol..

[72] Molina-Jiménez M.F., Sánchez-Reus M.I., Andres D., Cascales M., Benedi J. (2004). Neuroprotective effect of fraxetin and myricetin against rotenone-induced apoptosis in neuroblastoma cells. Brain Res..

[73] Sánchez-Reus M.I., Peinado I.I., Molina-Jiménez M.F., Benedí J. (2005). Fraxetin prevents rotenone-induced apoptosis by induction of endogenous glutathione in human neuroblastoma cells. Neurosci. Res..

[74] Li Y., Li J., Li Y., Wang X.X., Cao A.C. (2013). Antimicrobial Constituents of the Leaves of *Mikania micrantha* H. B. K. PLoS ONE.

[75] Liu J., Zhao Y., Shi Z., Bai Y. (2019). Antitumor effects of helenalin in doxorubicin-resistant leukemia cells are mediated via mitochondrial mediated apoptosis, loss of mitochondrial membrane potential, inhibition of cell migration and invasion and downregulation of PI3-kinase/AKT/m-TOR signa. J. Buon.

[76] Moyo P., Kunyane P., Selepe M.A., Eloff J.N., Niemand J., Louw A.I., Maharaj V.J., Birkholtz L.M. (2019). Bioassay-guided isolation and identification of gametocytocidal compounds from Artemisia afra (Asteraceae). Malar. J..

[77] Ramos A., Rivero R., Visozo A., Piloto J., García A. (2002). Parthenin, a sesquiterpene lactone of Parthenium hysterophorus L. is a high toxicity clastogen. Mutat. Res.-Genet. Toxicol. Environ. Mutagen..

[78] Takeda S., Matsuo K., Yaji K., Okajima-Miyazaki S., Harada M., Miyoshi H., Okamoto Y., Amamoto T., Shindo M., Omiecinski C.J. (2011). (-)-Xanthatin selectively induces GADD45γ and stimulates caspase-independent cell death in human breast cancer MDA-MB-231 cells. Chem. Res. Toxicol..

[79] Wu Q., Qin Z., Kuca K., You L., Zhao Y., Liu A., Musilek K., Chrienova Z., Nepovimova E., Oleksak P. (2020). An update on T-2 toxin and its modified forms: Metabolism, immunotoxicity mechanism, and human exposure assessment. Arch. Toxicol..

[80] Pai J.T., Hsu C.Y., Hua K.T., Yu S.Y., Huang C.Y., Chen C.N., Liao C.H., Weng M.S. (2015). NBM-T-BBX-OS01, semisynthesized from osthole, induced G1 growth arrest through HDAC6 inhibition in lung cancer cells. Molecules.

[81] Tang D.Z., Hou W., Zhou Q., Zhang M., Holz J., Sheu T.J., Li T.F., Cheng S.D., Shi Q., Harris S.E. (2010). Osthole stimulates osteoblast differentiation and bone formation by activation of β-catenin-BMP signaling. J. Bone Miner. Res..

[82] You L., Feng S., An R., Wang X. (2009). Osthole: A Promising Lead Compound for Drug Discovery from a Traditional Chinese Medicine (TCM). Nat. Prod. Commun..

[83] Balaji N.V., Ramani M.V., Viana A.G., Sanglard L.P., White J., Mulabagal V., Lee C., Gana T.J., Egiebor N.O., Subbaraju G.V. (2015). Design, synthesis and in vitro cell-based evaluation of the anti-cancer activities of hispolon analogs. Bioorganic Med. Chem..

[84] Han Y.H., Park W.H. (2010). Pyrogallol-induced calf pulmonary arterial endothelial cell death via caspase-dependent apoptosis and GSH depletion. Food Chem. Toxicol..

[85] Suh D.Y., Han Y.N., Han B.H. (1996). Maltol, an antioxidant component of Korean red ginseng, shows little prooxidant activity. Arch. Pharm. Res..

[86] Chemical Entities of Biological Interest Resveratrol CHEBI:27881. https://www.ebi.ac.uk/chebi/searchId.do?chebiId=CHEBI:27881.

[87] Yang C.J., Wang C.S., Hung J.Y., Huang H.W., Chia Y.C., Wang P.H., Weng C.F., Huang M.S. (2009). Pyrogallol induces G2-M arrest in human lung cancer cells and inhibits tumor growth in an animal model. Lung Cancer.

[88] Srivastava V., Darokar M.P., Fatima A., Kumar J.K., Chowdhury C., Saxena H.O., Dwivedi G.R., Shrivastava K., Gupta V., Chattopadhyay S.K. (2007). Synthesis of diverse analogues of Oenostacin and their antibacterial activities. Bioorganic Med. Chem..

[89] Andrioli W.J., Conti R., Araújo M.J., Zanasi R., Cavalcanti B.C., Manfrim V., Toledo J.S., Tedesco D., De Moraes M.O., Pessoa C. (2014). Mycoleptones A-C and polyketides from the endophyte mycoleptodiscus indicus. J. Nat. Prod..

[90] Joo S.H., Lee S.C., Kim S.K. (2004). UV absorbent, marmesin, from the bark of Thanakha, *Hesperethusa crenulata* L.. J. Plant Biol..

[91] National Center for Biotechnology Information PubChem Compound Summary for CID 5282965, 9,10,13-Trihydroxy-11-octadecenoic Acid. https://pubchem.ncbi.nlm.nih.gov/compound/5282965.

[92] National Center for Biotechnology Information PubChem Bioassay Record for AID 2300, Source: The Scripps Research Institute Molecular Screening Center. https://pubchem.ncbi.nlm.nih.gov/bioassay/2300.

[93] National Center for Biotechnology Information PubChem Compound Summary for CID 1052, Pyridoxamine. https://pubchem.ncbi.nlm.nih.gov/compound/Pyridoxamine.

[94] Kinoshita G., Nakamura F., Furuhata Y. (1987). Inhibitory effects of Saposhnikovia root on CNS functions and peptic ulcers. J. Med. Pharm. Soc. Wakan-Yaku.

[95] Chen N., Wu Q., Chi G., Soromou L.W., Hou J., Deng Y., Feng H. (2013). Prime-O-glucosylcimifugin attenuates lipopolysaccharide-induced acute lung injury in mice. Int. Immunopharmacol..

[96] Chang C.Z., Wu S.C., Kwan A.L., Lin C.L. (2015). 4’-O-β-D-glucosyl-5-O-methylvisamminol, an active ingredient of *Saposhnikovia divaricata*, attenuates high-mobility group box 1 and subarachnoid hemorrhage-induced vasospasm in a rat model. Behav. Brain Funct..

[97] National Center for Biotechnology Information PubChem Compound Summary for CID 76318697, Yadanzioside D. https://pubchem.ncbi.nlm.nih.gov/compound/Yadanzioside-D.

[98] National Center for Biotechnology Information PubChem Bioassay Record for AID 2330, Source: Broad Institute. https://pubchem.ncbi.nlm.nih.gov/bioassay/2330.

[99] National Center for Biotechnology Information PubChem Compound Summary for CID 5283137, Thromboxane B2. https://pubchem.ncbi.nlm.nih.gov/compound/5283137.

[100] National Center for Biotechnology Information PubChem Compound Summary for CID 5280888, 6-Keto-Prostaglandin F1alpha. https://pubchem.ncbi.nlm.nih.gov/compound/5280888.

[101] Chen B.H., Wu P.Y., Chen K.M., Fu T.F., Wang H.M., Chen C.Y. (2009). Antiallergic potential on RBL-2H3 cells of some phenolic constituents of Zingiber officinale (ginger). J. Nat. Prod..

[102] Aun L.L., Azmi M.N., Ibrahim H., Awang K., Nagoor N.H. (2011). 1′S-1′-acetoxyeugenol acetate: A novel phenylpropanoid from Alpinia conchigera enhances the apoptotic effects of paclitaxel in MCF-7 cells through NF-κB inactivation. Anticancer Drugs.

[103] National Center for Biotechnology Information PubChem Compound Summary for CID 28619, Precocene I. https://pubchem.ncbi.nlm.nih.gov/compound/Precocene-I.

[104] Musthafa K.S., Voravuthikunchai S.P. (2015). Anti-virulence potential of eugenyl acetate against pathogenic bacteria of medical importance. Antonie van Leeuwenhoek. Int. J. Gen. Mol. Microbiol..

[105] Kuca K., Dohnal V., Jezkova A., Jun D. (2008). Metabolic Pathways of T-2 Toxin. Curr. Drug Metab..

[106] Lin H.R. (2012). Sesquiterpene lactones from Tithonia diversifolia act as peroxisome proliferator-activated receptor agonists. Bioorganic Med. Chem. Lett..

[107] Emond J.P., Lacombe L., Caron P., Turcotte V., Simonyan D., Aprikian A., Saad F., Carmel M., Chevalier S., Guillemette C. (2021). Urinary oestrogen steroidome as an indicator of the risk of localised prostate cancer progression. Br. J. Cancer.

[108] Li S., Chen Y., Xie L., Meng Y., Zhu L., Chu H., Gu D., Zhang Z., Du M., Wang M. (2020). Sex hormones and genetic variants in hormone metabolic pathways associated with the risk of colorectal cancer. Environ. Int..

[109] Zhao F., Hao Z., Zhong Y., Xu Y., Guo M., Zhang B., Yin X., Li Y., Zhou X. (2021). Discovery of breast cancer risk genes and establishment of a prediction model based on estrogen metabolism regulation. BMC Cancer.

[110] National Center for Biotechnology Information PubChem Compound Summary for CID 23205, Helenalin. https://pubchem.ncbi.nlm.nih.gov/compound/Helenalin.

[111] Huang D.-N., Wang S., Sooranna S.R., Miao J.-H. (2021). The Efficacy of Natural Bioactive Compounds for the Treatment of Nasopharyngeal Carcinoma. Mini-Rev. Med. Chem..

[112] Picman J., Picman A.K. (1985). Treatment of dermatitis from parthenin. Contact Dermat..

[113] Huang W.J., Chen C.C., Chao S.W., Lee S.S., Hsu F.L., Lu Y.L., Hung M.F., Chang C.I. (2010). Synthesis of n-hydroxycinnamides capped with a naturally occurring moiety as inhibitors of histone deacetylase. ChemMedChem.

[114] Zhang C., Ning D., Pan J., Chen C., Gao C., Ding Z., Jiang F., Li M. (2021). Anti-Inflammatory Effect Fraction of Bletilla striata and Its Protective Effect on LPS-Induced Acute Lung Injury. Mediat. Inflamm..

[115] Pinkhien T., Petpiroon N., Sritularak B., Chanvorachote P. (2017). Batatasin III inhibits migration of human lung cancer cells by suppressing epithelial to mesenchymal transition and FAK-AKT signals. Anticancer Res..

[116] Sharma S., Nitya A. (1997). Natural products. Pharmacochemistry Library.

[117] Gaich T., Mulzer J. (2012). 2.7 Chiral Pool Synthesis: Starting from Terpenes. Comprehensive Chirality.

[118] Dhakal D., Han J.M., Mishra R., Pandey R.P., Kim T.S., Rayamajhi V., Jung H.J., Yamaguchi T., Sohng J.K. (2020). Characterization of Tailoring Steps of Nargenicin A1 Biosynthesis Reveals a Novel Analogue with Anticancer Activities. ACS Chem. Biol..

[119] Coppock R.W., Christian R.G., Jacobsen B.J. (2018). Aflatoxins. Veterinary Toxicology: Basic and Clinical Principles.

[120] Agahi F., Juan-García A., Font G., Juan C. (2021). Study of enzymatic activity in human neuroblastoma cells SH-SY5Y exposed to zearalenone’s derivates and beauvericin. Food Chem. Toxicol..

[121] MeSH Pharmacological Classification Antimetabolites, Antineoplastic-MeSH-NCBI. https://www.ncbi.nlm.nih.gov/mesh/68000964.

[122] National Center for Biotechnology Information PubChem Compound Summary for CID 3840, Kojic Acid. https://pubchem.ncbi.nlm.nih.gov/compound/Kojic-acid.

[123] Chemical Entities of Biological Interest Ferulic acid CHEBI:17620. https://www.ebi.ac.uk/chebi/searchId.do?chebiId=CHEBI:17620.

[124] Chemical Entities of Biological Interest Scoparone CHEBI:9055. https://www.ebi.ac.uk/chebi/searchId.do?chebiId=CHEBI:9055.

[125] Chemical Entities of Biological Interest Fraxetin CHEBI:5169. https://www.ebi.ac.uk/chebi/searchId.do?chebiId=CHEBI:5169.

[126] Michalak I., Chojnacka K. (2015). Algae as production systems of bioactive compounds. Eng. Life Sci..

[127] Leandro A., Pereira L., Gonçalves A.M.M. (2019). Diverse Applications of Marine Macroalgae. Mar. Drugs.

[128] Zhang G., Li J., Zhu T., Gu Q., Li D. (2016). Advanced tools in marine natural drug discovery. Curr. Opin. Biotechnol..

[129] Suleria H.A.R., Gobe G., Masci P., Osborne S.A. (2016). Marine bioactive compounds and health promoting perspectives; innovation pathways for drug discovery. Trends Food Sci. Technol..

[130] Sigwart J.D., Blasiak R., Jaspars M., Jouffray J.-B., Tasdemir D. (2021). Unlocking the potential of marine biodiscovery. Nat. Prod. Rep..

[131] Petras D., Koester I., Da Silva R., Stephens B.M., Haas A.F., Nelson C.E., Kelly L.W., Aluwihare L.I., Dorrestein P.C. (2017). High-resolution liquid chromatography tandem mass spectrometry enables large scale molecular characterization of dissolved organic matter. Front. Mar. Sci..

[132] Sogin E.M., Putnam H.M., Nelson C.E., Anderson P., Gates R.D. (2017). Correspondence of coral holobiont metabolome with symbiotic bacteria, archaea and Symbiodinium communities. Environ. Microbiol. Rep..

[133] Ulrich E.M., Sobus J.R., Grulke C.M., Richard A.M., Newton S.R., Strynar M.J., Mansouri K., Williams A.J. (2019). EPA’s non-targeted analysis collaborative trial (ENTACT): Genesis, design, and initial findings. Anal. Bioanal. Chem..

[134] Hollender J., Schymanski E.L., Singer H.P., Ferguson P.L. (2017). Nontarget Screening with High Resolution Mass Spectrometry in the Environment: Ready to Go?. Environ. Sci. Technol..

[135] Marshall A.G., Hendrickson C.L., Jackson G.S. (1998). Fourier transform ion cyclotron resonance mass spectrometry: A primer. Mass Spectrom. Rev..

[136] Nikolaev E.N., Kostyukevich Y.I., Vladimirov G.N. (2016). Fourier transform ion cyclotron resonance (FT ICR) mass spectrometry: Theory and simulations. Mass Spectrom. Rev..

[137] Zark M., Christoffers J., Dittmar T. (2017). Molecular properties of deep-sea dissolved organic matter are predictable by the central limit theorem: Evidence from tandem FT-ICR-MS. Mar. Chem..

[138] Hawkes J.A., Patriarca C., Sjöberg P.J.R., Tranvik L.J., Bergquist J. (2018). Extreme isomeric complexity of dissolved organic matter found across aquatic environments. Limnol. Oceanogr. Lett..

[139] Regal A.L., Alves V., Gomes R., Matos J., Bandarra N.M., Afonso C., Cardoso C. (2020). Drying process, storage conditions, and time alter the biochemical composition and bioactivity of the anti-greenhouse seaweed Asparagopsis taxiformis. Eur. Food Res. Technol..

[140] Nunes N., Ferraz S., Valente S., Barreto M.C., Pinheiro de Carvalho M.A.A. (2017). Biochemical composition, nutritional value, and antioxidant properties of seven seaweed species from the Madeira Archipelago. J. Appl. Phycol..

[141] Jard G., Marfaing H., Carrère H., Delgenes J.P., Steyer J.P., Dumas C. (2013). French Brittany macroalgae screening: Composition and methane potential for potential alternative sources of energy and products. Bioresour. Technol..

[142] Bogaert K.A., Delva S., De Clerck O. (2020). Concise review of the genus Dictyota J.V. Lamouroux. J. Appl. Phycol..

[143] Zubia M., Robledo D., Freile-Pelegrin Y. (2007). Antioxidant activities in tropical marine macroalgae from the Yucatan Peninsula, Mexico. J. Appl. Phycol..

[144] Vega J., Álvarez-Gómez F., Güenaga L., Figueroa F.L., Gómez-Pinchetti J.L. (2020). Antioxidant activity of extracts from marine macroalgae, wild-collected and cultivated, in an integrated multi-trophic aquaculture system. Aquaculture.

[145] Connan S., Goulard F., Stiger V., Deslandes E., Gall E.A. (2004). Interspecific and temporal variation in phlorotannin levels in an assemblage of brown algae. Bot. Mar..

[146] Celis-Plá P.S.M., Korbee N., Gómez-Garreta A., Figueroa F.L. (2014). Seasonal photoacclimation patterns in the intertidal macroalga *Cystoseira tamariscifolia* (Ochrophyta). Sci. Mar..

[147] Celis-Plá P.S.M., Brown M.T., Santillán-Sarmiento A., Korbee N., Sáez C.A., Figueroa F.L. (2018). Ecophysiological and metabolic responses to interactive exposure to nutrients and copper excess in the brown macroalga *Cystoseira tamariscifolia*. Mar. Pollut. Bull..

[148] Schneider G., Figueroa F.L., Vega J., Chaves P., Álvarez-Gómez F., Korbee N., Bonomi-Barufi J. (2020). Photoprotection properties of marine photosynthetic organisms grown in high ultraviolet exposure areas: Cosmeceutical applications. Algal Res..

[149] Bridi R., Troncoso M.J., Folch-Cano C., Fuentes J., Speisky H., López-Alarcón C. (2014). A Polyvinylpolypyrrolidone (PVPP)-Assisted Folin–Ciocalteu Assay to Assess Total Phenol Content of Commercial Beverages. Food Anal. Methods.

[150] Parys S., Rosenbaum A., Kehraus S., Reher G., Glombitza K.-W., König G.M. (2007). Evaluation of Quantitative Methods for the Determination of Polyphenols in Algal Extracts. J. Nat. Prod..

[151] Kumar M., Kuzhiumparambil U., Pernice M., Jiang Z., Ralph P.J. (2016). Metabolomics: An emerging frontier of systems biology in marine macrophytes. Algal Res..

[152] De Paula J.C., Cavalcanti D.N., Yoneshigue-Valentin Y., Teixeira V.L. (2012). Diterpenes from marine brown alga *Dictyota guineensis* (Dictyotaceae, Phaeophyceae). Rev. Bras. Farmacogn..

[153] Hamid S.S., Wakayama M., Ichihara K., Sakurai K., Ashino Y., Kadowaki R., Soga T., Tomita M. (2019). Metabolome profiling of various seaweed species discriminates between brown, red, and green algae. Planta.

[154] Gaubert J., Greff S., Thomas O.P., Payri C.E. (2019). Metabolomic variability of four macroalgal species of the genus Lobophora using diverse approaches. Phytochemistry.

[155] Nylund G.M., Weinberger F., Rempt M., Pohnert G. (2011). Metabolomic assessment of induced and activated chemical defence in the invasive red alga gracilaria vermiculophylla. PLoS ONE.

[156] Dixit D., Reddy C.R.K., Trivedi M.H., Gadhavi D.K. (2020). Non-targeted metabolomics approach to assess the brown marine macroalga *Dictyota dichotoma* as a functional food using liquid chromatography with mass spectrometry. Sep. Sci. Plus.

[157] Mateos R., Pérez-Correa J., Domínguez H. (2020). Bioactive properties of marine phenolics. Mar. Drugs.

[158] Santos S.A.O., Félix R., Pais A.C.S., Rocha S.M., Silvestre A.J.D. (2019). The Quest for Phenolic Compounds from Macroalgae: A Review of Extraction and Identification Methodologies. Biomolecules.

[159] Pinto D.C.G.A., Lesenfants M.L., Rosa G.P., Barreto M.C., Silva A.M.S., Seca A.M.L. (2022). GC-and UHPLC-MS Profiles as a Tool to Valorize the Red Alga *Asparagopsis armata*. Appl. Sci..

[160] Zhong B., Robinson N.A., Warner R.D., Barrow C.J., Dunshea F.R., Suleria H.A.R. (2020). LC-ESI-QTOF-MS/MS Characterization of Seaweed Phenolics and Their Antioxidant Potential. Mar. Drugs.

[161] Chauhan V., Chauhan A. (2006). Oxidative stress in Alzheimer’s disease. Pathophysiology.

[162] Thanan R., Oikawa S., Hiraku Y., Ohnishi S., Ma N., Pinlaor S., Yongvanit P., Kawanishi S., Murata M., Thanan R. (2014). Oxidative Stress and Its Significant Roles in Neurodegenerative Diseases and Cancer. Int. J. Mol. Sci..

[163] Blesa J., Trigo-Damas I., Quiroga-Varela A., Jackson-Lewis V.R. (2015). Oxidative stress and Parkinson’s disease. Front. Neuroanat..

[164] Siti H.N., Kamisah Y., Kamsiah J. (2015). The role of oxidative stress, antioxidants and vascular inflammation in cardiovascular disease (a review). Vascul. Pharmacol..

[165] Félix R., Dias P., Félix C., Cerqueira T., Andrade P.B., Valentão P., Lemos M.F.L. (2021). The biotechnological potential of *Asparagopsis armata*: What is known of its chemical composition, bioactivities and current market?. Algal Res..

[166] Mekinić I.G., Šimat V., Botić V., Crnjac A., Smoljo M., Soldo B., Ljubenkov I., Čagalj M., Skroza D. (2021). Bioactive phenolic metabolites from adriatic brown algae *Dictyota dichotoma* and padina pavonica (Dictyotaceae). Foods.

[167] Martins C.D.L., Ramlov F., Nocchi Carneiro N.P., Gestinari L.M., dos Santos B.F., Bento L.M., Lhullier C., Gouvea L., Bastos E., Horta P.A. (2013). Antioxidant properties and total phenolic contents of some tropical seaweeds of the Brazilian coast. J. Appl. Phycol..

[168] El-Shenody R.A., Ashour M., Ghobara M.M.E. (2019). Evaluating the chemical composition and antioxidant activity of three Egyptian seaweeds: *Dictyota dichotoma*, *Turbinaria decurrens*, and *Laurencia obtusa*. Braz. J. Food Technol..

[169] Platsidaki E., Dessinioti C. (2018). Recent advances in understanding *Propionibacterium acnes* (*Cutibacterium acnes*) in acne. F1000Research.

[170] Shanmughapriya S., Manilal A., Sujith S., Selvin J., Kiran G.S., Natarajaseenivasan K. (2008). Antimicrobial activity of seaweeds extracts against multiresistant pathogens. Ann. Microbiol..

[171] Tuney I., Cadirci B.H., Unal D., Sukatar A. (2007). Locational and organic solvent variation in antimicrobial activities of crude extracts of marine algae from the coast of Izmir (Turkey). Fresenius Environ. Bull..

[172] Lee M.S., Yim M.-J., Lee J.M., Lee D.-S., Kim M.-Y., Eom S.-H. (2021). In vitro Antimicrobial Activities of Edible Seaweeds Extracts Against *Cutibacterium acnes*. Korean J. Fish. Aquat. Sci..

[173] Pineau R.M., Hanson S.E., Lyles J.T., Quave C.L. (2019). Growth inhibitory activity of callicarpa americana leaf extracts against *Cutibacterium acnes*. Front. Pharmacol..

[174] De Canha M.N., Komarnytsky S., Langhansova L., Lall N. (2020). Exploring the Anti-Acne Potential of Impepho [Helichrysum odoratissimum (L.) Sweet] to Combat *Cutibacterium acnes* Virulence. Front. Pharmacol..

[175] Ina-Salwany M.Y., Al-saari N., Mohamad A., Mursidi F.A., Mohd-Aris A., Amal M.N.A., Kasai H., Mino S., Sawabe T., Zamri-Saad M. (2019). Vibriosis in Fish: A Review on Disease Development and Prevention. J. Aquat. Anim. Health.

[176] Fernández-Álvarez C., Santos Y. (2018). Identification and typing of fish pathogenic species of the genus *Tenacibaculum*. Appl. Microbiol. Biotechnol..

[177] Shannon E., Abu-Ghannam N. (2016). Antibacterial Derivatives of Marine Algae: An Overview of Pharmacological Mechanisms and Applications. Mar. Drugs.

[178] Cardoso S.L., Costa C.S.D., Nishikawa E., da Silva M.G.C., Vieira M.G.A. (2017). Biosorption of toxic metals using the alginate extraction residue from the brown algae *Sargassum filipendula* as a natural ion-exchanger. J. Clean. Prod..

[179] Varaprasad K., Jayaramudu T., Kanikireddy V., Toro C., Sadiku E.R. (2020). Alginate-based composite materials for wound dressing application:A mini review. Carbohydr. Polym..

[180] Wang Y., Xing M., Cao Q., Ji A., Liang H., Song S. (2019). Biological Activities of Fucoidan and the Factors Mediating Its Therapeutic Effects: A Review of Recent Studies. Mar. Drugs.

[181] Jönsson M., Allahgholi L., Sardari R.R.R., Hreggvidsson G.O., Nordberg Karlsson E. (2020). Extraction and Modification of Macroalgal Polysaccharides for Current and Next-Generation Applications. Molecules.

[182] Lee K.W., Lee J., Park S.I., Shin M.S. (2021). Antimicrobial, Antioxidative, Elastase and Tyrosinase Inhibitory Effect of Supercritical and Hydrothermal *Halopteris scoparia* Extract. Turk. J. Comput. Math. Educ..

[183] Mercado J.M., Gómez-Jakobsen F., Korbee N., Aviles A., Bonomi-Barufi J., Muñoz M., Reul A., Figueroa F.L. (2022). Analyzing environmental factors that favor the growth of the invasive brown macroalga *Rugulopteryx okamurae* (Ochrophyta): The probable role of the nutrient excess. Mar. Pollut. Bull..

[184] Lourenço S.O., Barbarino E., De-Paula J.C., Pereira L.O.D.S., Lanfer Marquez U.M. (2002). Amino acid composition, protein content and calculation of nitrogen-to-protein conversion factors for 19 tropical seaweeds. Phycol. Res..

[185] DuBois M., Gilles K.A., Hamilton J.K., Rebers P.A., Smith F. (1956). Colorimetric Method for Determination of Sugars and Related Substances. Anal. Chem..

[186] Folch J., Lees M., Sloan Stanley G. (1957). A simple method for the isolation and purification of total lipids from animal tissues. J. Biol. Chem..

[187] Singleton V., Rossi J. (1965). Colorimetry of total phenolics with phosphomolybdic-phosphotungstic acid reagents. Am. J. Enol. Vitic..

[188] Van Alstyne K.L. (1995). Comparison of three methods for quantifying brown algal polyphenolic compounds. J. Chem. Ecol..

[189] Hansman R.L., Dittmar T., Herndl G.J. (2015). Conservation of dissolved organic matter molecular composition during mixing of the deep water masses of the northeast Atlantic Ocean. Mar. Chem..

[190] Osterholz H., Singer G., Wemheuer B., Daniel R., Simon M., Niggemann J., Dittmar T. (2016). Deciphering associations between dissolved organic molecules and bacterial communities in a pelagic marine system. ISME J..

[191] Merder J., Freund J.A., Feudel U., Hansen C.T., Hawkes J.A., Jacob B., Klaproth K., Niggemann J., Noriega-Ortega B.E., Osterholz H. (2020). ICBM-OCEAN: Processing Ultrahigh-Resolution Mass Spectrometry Data of Complex Molecular Mixtures. Anal. Chem..

[192] Merder J., Freund J.A., Feudel U., Niggemann J., Singer G., Dittmar T. (2020). Improved mass accuracy and isotope confirmation through alignment of ultrahigh-resolution mass spectra of complex natural mixtures. Anal. Chem..

[193] Koch B.P., Dittmar T. (2006). From mass to structure: An aromaticity index for high-resolution mass data of natural organic matter. Rapid Commun. Mass Spectrom..

[194] (2010). The Pallets Projects Welcome to Flask—Flask Documentation (2.0.x). https://flask.palletsprojects.com/en/2.0.x/.

[195] Reitz K., Benfield C., Cordasco I., Prewitt N., Larson S.M. (2021). (2022) Requests: HTTP for humans. A Kenneth Reitz Project. https://docs.python-requests.org/en/master/.

[196] Kim S., Chen J., Cheng T., Gindulyte A., He J., He S., Li Q., Shoemaker B.A., Thiessen P.A., Yu B. (2021). PubChem in 2021: New data content and improved web interfaces. Nucleic Acids Res..

[197] Haug K., Cochrane K., Nainala V.C., Williams M., Chang J., Jayaseelan K.V., O’Donovan C. (2020). MetaboLights: A resource evolving in response to the needs of its scientific community. Nucleic Acids Res..

[198] Kim S., Thiessen P.A., Cheng T., Yu B., Bolton E.E. (2018). An update on PUG-REST: RESTful interface for programmatic access to PubChem. Nucleic Acids Res..

[199] European Bioinformatics Institute MetaboLights RESTful WebService. https://www.ebi.ac.uk/metabolights/ws/api/spec.html#!/spec.

[200] Re R., Pellegrini N., Proteggente A., Pannala A., Yang M., Rice-Evans C. (1999). Antioxidant activity applying an improved ABTS radical cation decolorization assay. Free Radic. Biol. Med..

[201] Brand-Williams W., Cuvelier M.E., Berset C. (1995). Use of a free radical method to evaluate antioxidant activity. LWT-Food Sci. Technol..

[202] García-Márquez J., Barany A., Ruiz Á.B., Costas B., Arijo S., Mancera J.M. (2021). Antimicrobial and toxic activity of citronella essential oil (*Cymbopogon nardus*), and its effect on the growth and metabolism of gilthead seabream (sparus aurata l.). Fishes.

[203] Oksanen J., Simpson G.L., Blanchet F.G., Kindt R., Legendre P., Minchin P.R., Al E. (2022). Community Ecology Package [R package vegan version 2.6-4]. https://cran.r-project.org/web/packages/vegan/vegan.pdf.

