# Supplementary material for: Molecular Diversity and Biochemical Content in Two Invasive Alien Species: Looking for Chemical Similarities and Bioactivities
Source: Mar Drugs. 2022 Dec 22;21(1):5. doi: 10.3390/md21010005 (PMC9861339; doi:10.3390/md21010005)

Table S1. Molecular composition and presence of molecular groups (percentage with respect to the total of formulas) for the different extractions in different solvents: water ($_{d}H_2O$), $_{d}H_2O$:Ethanol (EtOH) (1:1), $_{d}H_2O$:EtOH (1:4), $_{d}H_2O$:Methanol (MeOH) (1:1), $_{d}H_2O$:MeOH (1:4), from the biomass of *Asparagopsis armata* and *Rugulopteryx okamurae*. Values are expressed as average ± standard deviation (SD) (n=3).

	**Sample Name**	**Total Number of formulae**	**% of exclusive formulae**	**Aromatic**		**Highly unsaturated**		**Polyphenols**		**Unsaturated**			**Saturated**	
				**O rich**	**O poor**	**O rich**	**O poor**	**O rich**	**O poor**	**O rich**	**O poor**	**with N***	**O rich**	**O poor**
A. Armata	$_{d}H_2O$	320 ± 44	15	11 ± 3	26 ± 4	16 ± 2	12 ± 2	8 ± 1	0 ± 0	17 ± 2	17 ± 6	11 ± 2	0 ± 0	1 ± 0
	$_{d}H_2O$:EtOH (1:1)	315 ± 57	4	4 ± 1	11 ± 0	6 ± 1	22 ± 1	11 ± 2	1 ± 0	11 ± 1	40 ± 2	25 ± 3	2 ± 0	4 ± 0
	$_{d}H_2O$:EtOH (1:4)	286 ± 56	2	4 ± 0	14 ± 2	9 ± 1	21 ± 1	9 ± 1	1 ± 0	12 ± 1	35 ± 4	29 ± 2	2 ± 0	2 ± 0
	$_{d}H_2O$:MeOH (1:1)	279 ± 48	2	5 ± 0	13 ± 1	7 ± 0	20 ± 2	11 ± 2	0 ± 0	13 ± 0	37 ± 2	29 ± 1	2 ± 1	2 ± 0
	$_{d}H_2O$:MeOH (1:4)	447 ± 75	4	4 ± 1	12 ± 2	12 ± 4	35 ± 8	10 ± 1	0 ± 0	9 ± 1	25 ± 7	20 ± 7	1 ± 0	1 ± 1
R. Okamurae	$_{d}H_2O$	233 ± 12	6	5 ± 2	20 ± 5	8 ± 3	18 ± 2	10 ± 1	1 ± 0	10 ± 3	35 ± 14	5 ± 0	2 ± 1	1 ± 0
	$_{d}H_2O$:EtOH (1:1)	372 ± 20	5	2 ± 0	12 ± 1	5 ± 1	42 ± 1	9 ± 1	1 ± 0	7 ± 0	30 ± 2	6 ± 2	1 ± 0	1 ± 0
	$_{d}H_2O$:EtOH (1:4)	357 ± 87	4	1 ± 0	10 ± 2	3 ± 1	51 ± 4	10 ± 1	2 ± 0	8 ± 0	24 ± 4	9 ± 6	2 ± 0	1 ± 0
	$_{d}H_2O$:MeOH (1:1)	210 ± 48	1	2 ± 0	13 ± 3	6 ± 1	51 ± 3	8 ± 1	1 ± 0	6 ± 1	19 ± 5	3 ± 1	2 ± 0	0 ± 0
	$_{d}H_2O$:MeOH (1:4)	525 ± 108	3	1 ± 0	10 ± 1	3 ± 0	52 ± 0	8 ± 1	1 ± 0	8 ± 1	24 ± 1	5 ± 1	1 ± 0	1 ± 0

* Unsaturated with N are part already of the Unsaturated O rich and O poor groups, therefore, do not quantify as an independent group.

Table S2. Pearson coefficient (r) between the different variables analyzed in this work for the different extractions in different solvents: water ($_{d}H_2O$), $_{d}H_2O$:Ethanol (EtOH) (1:1), $_{d}H_2O$:EtOH (1:4), $_{d}H_2O$:Methanol (MeOH) (1:1), $_{d}H_2O$:MeOH (1:4), from the biomass of *Asparagopsis armata* and *Rugulopteryx okamurae*. Green: Positive correlation, Red: Negative correlations. **: p<0.01, *p<0.05.

	TPC	TPC (PVPP)	ABTS	DPPH	CHO	CHON	CHOS	CHOP	Arom. Or	Arom. Op	H satur. Or	H satur. Op	Unsatur. Or	Unsatur. Op	Unsatur. N	Satur. Or	Satur. Op
**TPC**	1,0000	,8785**	,7994**	,7717**	,9056**	-,8589**	-,6846**	,6083**	-,6584**	-,3526	-,6449**	,7277**	-,7148**	-,1164	-,6856**	-,0592	-,4858**
**TPC-PVPP**		1,0000	,5738**	,5782**	,8334**	-,8257**	-,5656**	,5909**	-,5615**	-,2981	-,5824**	,7023**	-,6195**	-,1940	-,6796**	-,0523	-,4978**
**ABTS**			1,0000	,9744**	,7805**	-,7114**	-,3926*	,2139	-,3711**	-,0455	-,3378	,3718*	-,5331**	-,0447	-,6353**	,0239	-,4522*
**DPPH**				1,0000	,7867**	-,7370**	-,3430	,2173	-,3156	,0271	-,3104	,3201	-,4993**	-,0477	-,6758**	,0226	-,4536*
**CHO**					1,0000	-,9012**	-,6435**	,5234**	-,6520**	-,3281	-,5244**	,7228**	-,7458**	-,1477	-,8036**	-,1188	-,5864**
**CHON**						1,0000	,3836*	-,6780**	,4071*	,1294	,3984*	-,6538**	,5573**	,3163	,9267**	,3162	,7011**
**CHOS**							1,0000	-,4949**	,8961**	,7176**	,6992**	-,7614**	,7245**	-,0894	,1918	-,0675	,2060
**CHOP**								1,0000	-,4741**	-,4132*	-,4325*	,7372**	-,3130	-,3449	-,5735**	-,4047*	-,5414**
**Arom. Or**									1,0000	,7987**	,7477**	-,7387**	,6449**	-,1810	,1991	-,0794	,1510
**Arom. Op**										1,0000	,6080**	-,5692**	,5148**	-,3648*	-,0638	,0721	-,0764
**H satur. Or**											1,0000	-,4623**	,5516**	-,4470*	,0702	-,1226	-,0824
**H satur. Op**												1,0000	-,6926**	-,4332*	-,5712**	-,1862	-,5631**
**Unsatur. Or**													1,0000	-,0605	,4486*	,1298	,2317
**Unsatur. Op**														1,0000	,5142**	,1517	,6412**
**Unsatur. N**															1,0000	,4064*	,7911**
**Satur. Or**																1,0000	,3727*
**Satur. Op**																	1,0000

TPC: Total phenolic compounds; Arom. Or: Aromatic oxygen rich; Arom. Op: Aromatic oxygen poor; H. satur. Or: Highly saturated oxygen rich; H. satur. Op: Highly saturated oxygen poor; Unsatur. Or: Unsaturated oxygen rich, Unsatur. Op: Unsaturated oxygen poor; Unsatur. N: Unsaturated Nitrogen; Satur. Or: Saturated oxygen rich; Satur. Op: Saturated oxygen poor.

Figure S1. ChemCrawler Interface (a) and an example of molecular search (b)

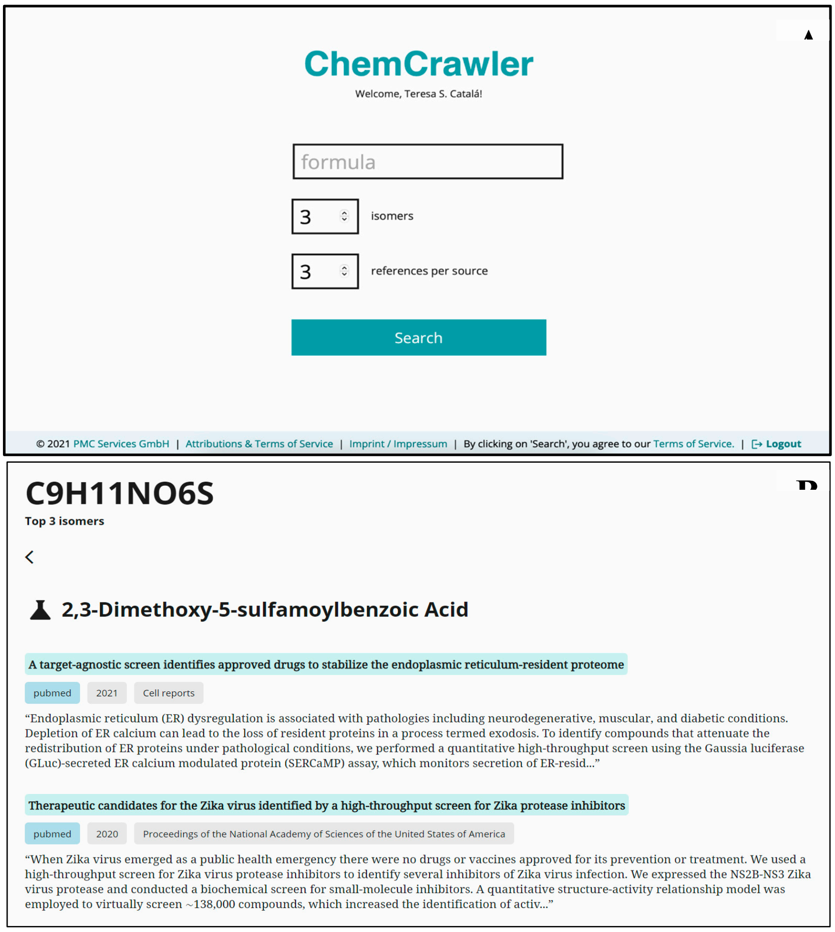

Supplement: Supplementary file 1 [file marinedrugs-21-00005-s001.zip › marinedrugs-2072760-supplementary.pdf]
